# Upregulation of EGFR signaling is correlated with tumor stroma remodeling and tumor recurrence in FGFR1-driven breast cancer

**DOI:** 10.1186/s13058-015-0649-1

**Published:** 2015-11-18

**Authors:** Xue B. Holdman, Thomas Welte, Kimal Rajapakshe, Adam Pond, Cristian Coarfa, Qianxing Mo, Shixia Huang, Susan G. Hilsenbeck, Dean P. Edwards, Xiang Zhang, Jeffrey M. Rosen

**Affiliations:** Graduate Program in Integrative Molecular and Biomedical Sciences, Baylor College of Medicine, One Baylor Plaza, Houston, TX 77030 USA; Department of Molecular and Cellular Biology, Baylor College of Medicine, One Baylor Plaza, Houston, TX 77030 USA; Lester and Sue Smith Breast Center, Baylor College of Medicine, One Baylor Plaza, Houston, TX 77030 USA; Duncan Cancer Center, Baylor College of Medicine, One Baylor Plaza, Houston, TX 77030 USA; Department of Pathology & Immunology, Baylor College of Medicine, One Baylor Plaza, Houston, TX 77030 USA

## Abstract

**Introduction:**

Despite advances in early detection and adjuvant targeted therapies, breast cancer is still the second most common cause of cancer mortality among women. Tumor recurrence is one of the major contributors to breast cancer mortality. However, the mechanisms underlying this process are not completely understood. In this study, we investigated the mechanisms of tumor dormancy and recurrence in a preclinical mouse model of breast cancer.

**Methods:**

To elucidate the mechanisms driving tumor recurrence, we employed a transplantable Wnt1/inducible fibroblast growth factor receptor (FGFR) 1 mouse mammary tumor model and utilized an FGFR specific inhibitor, BGJ398, to study the recurrence after treatment. Histological staining was performed to analyze the residual tumor cells and tumor stroma. Reverse phase protein array was performed to compare primary and recurrent tumors to investigate the molecular mechanisms leading to tumor recurrence.

**Results:**

Treatment with BGJ398 resulted in rapid tumor regression, leaving a nonpalpable mass of dormant tumor cells organized into a luminal and basal epithelial layer similar to the normal mammary gland, but surrounded by dense stroma with markedly reduced levels of myeloid-derived tumor suppressor cells (MDSCs) and decreased tumor vasculature. Following cessation of treatment the tumors recurred over a period of 1 to 4 months. The recurrent tumors displayed dense stroma with increased collagen, tenascin-C expression, and MDSC infiltration. Activation of the epidermal growth factor receptor (EGFR) pathway was observed in recurrent tumors, and inhibition of EGFR with lapatinib in combination with BGJ398 resulted in a significant delay in tumor recurrence accompanied by reduced stroma, yet there was no difference observed in initial tumor regression between the groups treated with BGJ398 alone or in combination with lapatinib.

**Conclusion:**

These studies have revealed a correlation between tumor recurrence and changes of stromal microenvironment accompanied by altered EGFR signaling.

**Electronic supplementary material:**

The online version of this article (doi:10.1186/s13058-015-0649-1) contains supplementary material, which is available to authorized users.

## Introduction

Tumor dormancy, a specific stage in cancer progression in which residual disease is present but remains asymptomatic, has been a major issue in cancer research for many years [1]. Possible mechanisms that have been suggested to contribute to tumor dormancy include: insufficient angiogenesis, an effective immune-suppressive response that keeps the cancer cells in check, and crosstalk with cells or proteins released in the microenvironment to arrest cancer cells in G0 stage [2]. Dormant cells, remaining undetectable over a prolonged period of time after the initial treatment, may exit from dormancy upon receiving stimuli, such as growth factors, cytokines, nutrients, or chemical agents, and re-enter the cell cycle to proliferate, eventually resulting in a life-threatening recurrence.

The stromal microenvironment has been increasingly recognized as a critical factor for cancer progression [1]. Changes in the stroma, which occur either during or after treatment, may facilitate tumor recurrence [3, 4]. In breast cancer, women with dense breasts detected by mammography have a two- to sixfold increase in their susceptibility to develop breast cancer [5] and breast cancers are thought to most likely arise from these dense tissues [6]. In fact, mammographic density, comprising epithelial and fibrous stromal tissues, is considered as a predictor of breast cancer outcome [7]. Moreover, changes in expression of certain genes in the mammary stroma are predictive markers in breast cancer pathogenesis [8–10]. Overall these studies indicate that the stroma is strongly associated with breast cancer progression.

There is therefore a need for the development of efficient therapeutic strategies for targeting the stromal microenvironment in breast cancer, especially to prevent tumor recurrence. However, this goal has been hampered due to paucity of preclinical models that can recapitulate breast cancer dormancy and recurrence [11]. Genetically engineered mouse models have provided one of the few approaches to study the mechanisms responsible for dormancy in vivo in the presence of an intact immune system and microenvironment. For example, recent studies performed in a doxycycline-regulatable erbB2-driven model have helped identify Notch signaling as important in tumor recurrence [12]. Furthermore, appropriate mouse models are a necessary prerequisite for testing new targeted therapies directed against the stromal microenvironment as well as the residual dormant cells. Although distant, as opposed to local, recurrence is clinically most relevant, studies of dormancy at distant sites are less tractable. Furthermore, understanding the mechanisms of local recurrence may help provide insights in developing therapeutic strategies for distant recurrence. In our studies, we employed a transplantable, genetically engineered Wnt1/ inducible fibroblast growth factor receptor 1 (iFGFR1; iR1) mouse mammary tumor model to study the recurrence of fibroblast growth factor receptor (FGFR)1-driven breast tumor. The FGFR signaling pathway plays a critical role in regulating normal mammary gland development and tissue homeostasis [13]. Dysregulation of FGFR signaling is associated with tumor recurrence in lung [14], bladder [13] and pancreatic cancer [15]. Analysis of copy number abnormalities has shown a consistently high level of amplification of chromosomal region 8p11 containing the FGFR1 coding region in early-stage breast cancers, resulting in overexpression of FGFR1 [13]. While FGFR1 alone resulted in very few palpable tumors with a long latency, FGFR1 is often amplified with other oncogenes such as c-MYC and CCND1 in breast cancer, which are downstream targets of the canonical Wnt signaling pathway [16]. Genetic analyses of mouse mammary tumor virus integration sites as well as functional studies using transgenic mouse models have provided considerable evidence supporting the cooperativity between the FGFR and Wnt pathways in mammary tumorigenesis [17–20]. Approximately 10 % of breast cancers have FGFR1 amplification and its amplification is associated with early relapse, poor survival and drug resistance [21]. Taken together, these findings suggest that FGFR1 is associated with tumor recurrence and targeting the FGFR1 signaling pathway might prevent tumor relapse.

In this study, treatment of mice bearing Wnt1/iR1 tumors with BGJ398 [22], a potent and selective inhibitor of the FGFR family of receptor tyrosine kinases, resulted in an altered collagen-enriched stroma observed both during tumor dormancy and recurrence. The phosphorylated epidermal growth factor receptor (EGFR) signaling pathway was upregulated in the recurrent tumors, and the combined inhibition of EGFR and FGFR1 signaling reduced the collagen-enriched stroma and significantly delayed tumor recurrence. Overall, these findings suggest that stroma remodeling during FGFR1 inhibitor treatment is important both for tumor dormancy and recurrence, and, furthermore, that recurrence can be delayed by inhibiting both FGFR1 and EGFR signaling.

## Methods and materials

### Kinase inhibitors

The inhibitor NVP-BGJ398 [22] was kindly provided by Dr. Diana Graus Porta (Novartis Institutes for Biomedical Research, Basel, Switzerland). NVP-BGJ398 was prepared as 10 mmol/L dimethyl sulfoxide (DMSO) stocks. The inhibitor lapatinib (free base) was purchased from LC Laboratories, Woburn, MA (; Cat#: L-4899). Lapatinib was prepared as 125 mg/ml DMSO stocks. Both inhibitors were diluted in the corresponding carrier for in vivo experiments.

### Animals, tumor transplant, and treatment

MMTV-Wnt1/iR1 mice were generated and characterized previously [20]. All mice were FVB background littermates. Mice were maintained in accordance with the NIH Guide for the Care and Use of Experimental Animals with approval from the Baylor College of Medicine Institutional Animal Care and Use Committee. Mice were injected intraperitoneally with 1 mg/kg B/B homodimerizer, a chemical dimerizer for the iFGFR model (Clontech, Mountain View, CA, every 3 days. Tumors were collected, dissected into small pieces and transplanted into the cleared fourth mammary fat pad of 3- to 4-week-old Fvb mice (Harlan Laboratories, Houston, TX. Following orthotopic transplantation, the B/B homodimerizer was injected 1 × 2 weeks after surgery to stimulate tumor growth. Tumors were measured and volume was calculated using the following formula: volume = height × ((diameter/2)^2^ × π).

Mice were randomly distributed into groups when tumors reached 300–600 mm^3^. Different groups were treated for the indicated times with different doses depending upon the experiment: vehicle (DMSO/D5W/PEG400 (1:2:2)), NVP-BGJ398 (per oral (p.o.), once daily, 30 mg/kg)), lapatinib (p.o., once daily, 150 mg/kg), and the combination of NVP-BGJ398 and lapatinib. Mice were sacrificed for residual tissue collection or left for future tumor recurrence.

### Immunohistochemistry, immunofluorescence, and quantification

All sections were pretreated using a sodium citrate antigen retrieval protocol as previously described [23]. Antibodies were used according to the manufacturer’s protocols (Additional file 1: Table S1).

Quantification of bromodeoxyuridine (BrdU), Ki67, cleaved caspase 3 (cc3) and S100A8 was obtained by counting at least three random sections. Positive staining was then quantified for each picture as a percentage of the total area of all blue 4′, 6-diamidino-2-phenylindole (DAPI) nuclear staining. At least a hundred cells per picture were counted using ImageJ software [24]. The percentage of vasculature was quantified using ImageJ software by analyzing the percentage area of positive vasculature in the total area [24] with at least five independent pictures for each group. All images showed in the manuscript are representative of at least three mice and at least three sections for each mouse analyzed.

### Masson’s trichrome staining

Tissue sections were deparaffinized, rehydrated with graded alcohols, fixed with Bouin’s solution (Sigma-Aldrich, The Woodlands, TX) overnight, washed in running tap water for 5 min and rinsed with distilled water. Thereafter, the sections were stained with Weigert Hematoxylin (Sigma-Aldrich, The Woodlands, TX) for 10 min and washed with distilled water for cell nuclei staining. Smooth muscle was stained red with Biebrich Scarlet-Acid-Fuschin (Sigma-Aldrich, The Woodlands, TX) for 10 min, followed by immersion in phosphomolybdic phosphotungstic acid (Sigma-Aldrich, The Woodlands, TX) for 15 min. Collagen was stained blue with Aniline Blue (Sigma-Aldrich) for 10 min and immersed in 1 % acetic acid for 5 min. Finally, the tissues were rehydrated with graded ethanol and xylene, and mounted with mounting medium. The percentage of collagen was quantified by ImageJ by analyzing the percentage area of positive collagen in the total area [24] with five independent pictures for each group.

### Immunoblot analysis and quantification

Pulverized, frozen tissues were resuspended in radioimmunoprecipitation assay buffer or tissue protein extraction buffer (T-PER; Life Technologies, Grand Island, NY, USA) plus phosphatase (Cell Signaling, Denvers, MA) and protease (Roche, Indianapolis, IN, USA) inhibitors. Tissue lysates were quantified using the bicinchoninic acid method (BCA; Pierce, Rockford, IL, USA), separated using SDS-PAGE, and transferred to PVDF membranes. All antibodies were used according to manufacturer’s protocols (Additional file 1: Table S1).

### Quantitative reverse transcription PCR

cDNA templates were generated using a High-Capacity RNA-to-cDNA™ Kit and 2 μg RNA per sample according to the manufacturer’s protocol (Invitrogen, Grand Island, NY). Quantitative PCRs were run using SYBR Green reagent (Applied Biosystems, Foster City, CA, USA) on a StepOnePlus thermocycler (Applied Biosystems), and fold changes were calculated using the comparative CT (ΔΔCT) method and StepOne software v2.0.1 (Applied Biosystems). Primer sequences for amphiregulin (Areg) are: forward 5′- GCC ATT ATG CAG CTG CTT TGG AGC -3′, reverse 5′- TGT TTT TCT TGG GCT TAA TCA CCT -3′.

### Reverse phase protein array analysis

Reverse phase protein array (RPPA) assays were carried out as described previously with minor modifications [25]. Protein lysates were prepared from tissue samples with tissue protein extraction reagent (TPER; Pierce) supplemented with 450 mM NaCl and a cocktail of protease and phosphatase inhibitors (Roche Life Science). Protein lysates at 0.5 mg/ml of total protein were denatured in SDS sample buffer (Life Technologies) containing 2.5 % 2-mercaptoethanol and treated at 100 °C for 8 min. The Aushon 2470 Arrayer (Aushon BioSystems, Billerica, MA, USA) with a 40-pin (185 μm) configuration was used to spot lysates onto nitrocellulose-coated slides (Grace Bio-labs, Bend, OR, USA) using an array format of 960 lysates/slide (2880 spots/slide) with each sample spotted as technical triplicates including test and control lysates.

Antibody labeling was performed at room temperature with an automated slide stainer Autolink 48 (Dako, Carpinteria, CA, USA). Slides were prepared for antibody labeling by blocking for 1 h with I-Block reagent (Applied Biosystems) followed by 15 min with Re-Blot reagent (Dako). After blocking, slides were loaded to the autostainer.

Each slide was incubated with primary antibody for 30 min followed by an appropriate biotinylated-secondary antibody for 15 min (goat anti-mouse IgG for mouse monoclonal primary antibodies, goat anti-rabbit IgG for rabbit primary antibodies, or rabbit anti-goat IgG for goat primary antibodies (Vector, Burlingame, CA, USA)). As a signal amplification step, slides were incubated for 15 min with Vectastain-ABC Streptavidin-Biotin Complex (Vector, PK-6100) followed by 15 min with TSA-plus Biotin Amp Reagent diluted at 1:250 (Perkin Elmer, Waltham, MA, USA). A fluorescent detection signal was generated by incubating slides for 15 min with a 1:50 dilution of LI-COR IRDye 680 Streptavidin (Odyssey, Lincoln, NE, USA). Total protein content of each spotted lysate was assessed by fluorescent staining for selected subset of slide with Sypro Ruby Protein Blot Stain according to the manufacturer’s instructions (Molecular Probes, Eugene, OR, USA). Fluorescent-labeled slides were scanned on a GenePix AL4200 scanner, and the images were analyzed with GenePix Pro 7.0 (Molecular Devices, Sunnyvale, CA). Total fluorescence signal intensities of each spot were obtained after subtraction of the local background signal for each slide, and were then normalized for variation in total protein, background and non-specific labeling using a group-based normalization method as described [26] with modification. For each spot on the array, the background-subtracted foreground signal intensity was subtracted by the corresponding signal intensity of the negative control slide (omission of primary antibody) and then normalized to the corresponding signal intensity of total protein for that spot. The distribution of the normalized data were summarized by descriptive statistics (e.g., mean, SD, range, median and quartiles) and tested for normality (e.g., Shapiro-Wilk and Shapiro-Francia test) using R statistical software. We determined significantly changed proteins between experimental groups by employing Student’s *t*-test (significant for *p* < 0.05) and requesting a fold change of at least 1.5 ×.

There are a total of 204 validated antibodies for RPPA. Of these, 132 antibodies detect total protein and 72 detect specific phosphorylated states of proteins known to be markers of protein activation states. Antibody validation for RRPA includes the selective detection of a single predominant protein band of the expected size by immunoblot assay of multiple known positive and negative tissues or cell lines and quality performance with the control lysates by RPPA assay. Each primary antibody is used at a pre-determined optimal dilution that generates appropriate differential signals across the range of control lysates. The validated antibodies represent proteins in various signaling pathways and cell functional groups including growth factor receptors, cell cycle, cell proliferation, apoptosis, EMT, stem cells, DNA damage, cell stress, autophagy, cytokines, protein translation and gene transcriptional activators and repressors. For a complete list of validated antibodies see https://www.bcm.edu/centers/cancer-center/research/shared-resources/antibody-based-proteomics.

### Gene signature analysis

The WNT signature is derived from “Additional file 5” in Huang et al. [27]. For each tumor sample, a WNT signature score is calculated as the sum of upregulated genes subtracted by the sum of downregulated genes. The score is then re-scaled linearly to [0, 1]. Tumors with FGFR genome copy number ≥4 and/or gene expression value ≥ mean (all samples) + SD (all samples) are defined as FGFR-amplified tumors. Survival analyses are performed using the survdiff function of the survival package in the R statistical software.

### Statistical analysis

The significance of fold changes for immunofluorescence/immunohistochemistry and reverse transcription PCR results was determined using student’s *t* tests and analysis of variance tests. All tests and graphical representation of data were performed using GraphPad Prism. For latency calculation, days were counted from the day when the treatment stopped to the day when recurrent tumors reach 100 mm^3^. Survival analysis was used to compare time to major tumor regression (defined as <20 % of baseline) and time to relapse (defined as regrowth >20 % of baseline, around 100 mm^3^). The generalized Wilcoxon test was used to test for differences. Shaded regions on the graphs show 95 % confidence regions.

### Consent statement

We confirm that this study did not involve human patients and no consent was required.

## Results

### Inhibiting FGFR1 leads to rapid regression of Wnt1/iR1 tumors

**Fig. 1:**
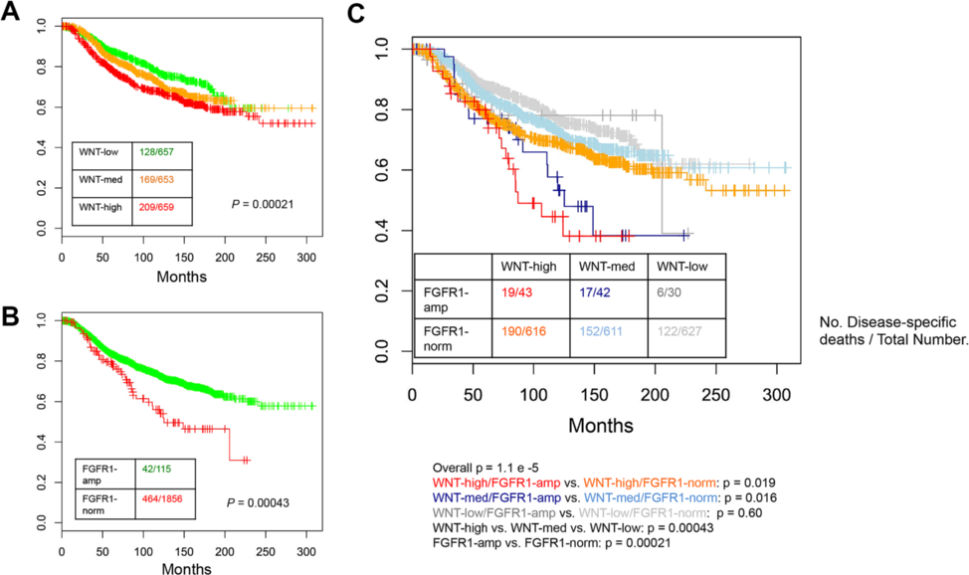
Human breast tumors with both FGFR1 amplification/overexpression and active WNT signaling exhibited the worst prognosis. **a** Kaplan-Meier curves show the disease-specific survival of patients in METABRIC dataset stratified by the statuses of WNT signature. WNT status is determined by ranking the continuous WNT score (sum of upregulated genes – sum of downregulated genes), and then separating all patients into three equal tertiles. **b**. The Kaplan-Meier curves show the disease-specific survival of patients in METABRIC dataset stratified by the status of FGFR1 amplification/overexpression. **c**. Kaplan-Meier curves show the disease-specific survival of patients in METABRIC dataset stratified by the status of FGFR1 amplification/overexpression and WNT signature. The number of events and total patients in each subset is indicated in parentheses. The *p* values are calculated based on log rank tests. *FGFR1* Fibroblast growth factor receptor 1

Previously, MMTV-Wnt1/iR1 mice were shown to develop tumors significantly faster than MMTV-Wnt1 alone. Additionally, the average tumor multiplicity was significantly greater in the Wnt1/iR1 bigenic mice compared to Wnt1 alone, and Wnt1/iR1 tumors grew significantly faster than the Wnt1 tumors [20]. Interestingly, it has been reported that in the iR1 model, activation of FGFR1 alone only resulted in increased lateral budding, alveolar hyperplasia, as well as increased macrophage recruitment to hyperplastic lesions, but very few palpable tumors developed in virgin mice even after very long latencies [28, 29]. The importance of cooperative effects between Wnt and FGFR1 signaling observed in mouse mammary tumor models is supported by our recent analysis of patient outcomes in human breast cancer. While either active WNT signaling or FGFR1 amplification/overexpression is associated with poor disease-specific survival (Fig. 1a and b), tumors with both pathways activated exhibited the worst prognosis, supporting that the two pathways may cooperate to drive tumor progression in human breast cancer (Fig. 1c).

**Fig. 2:**
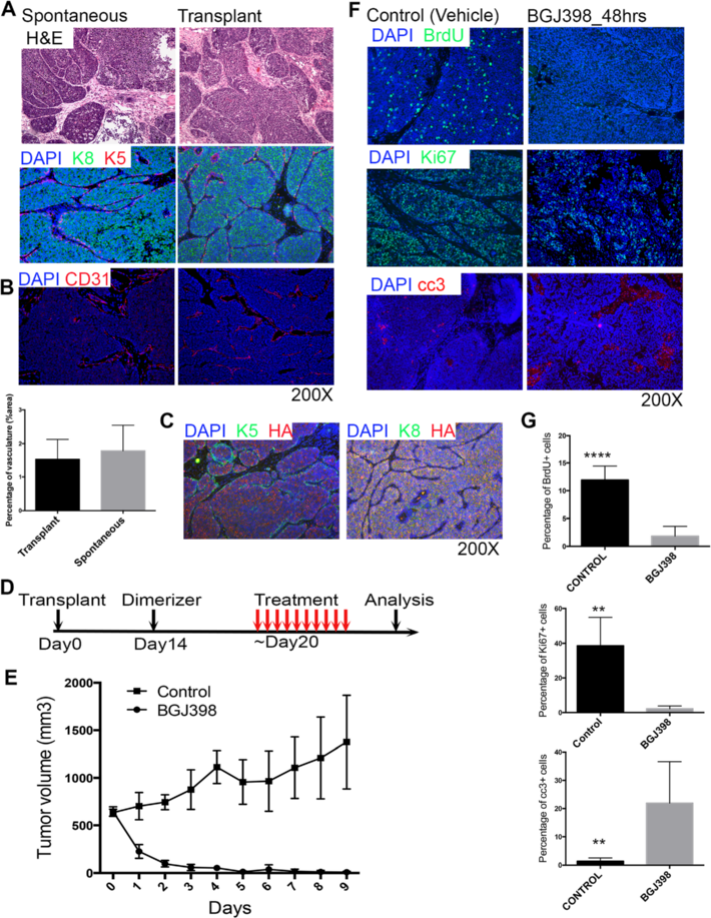
Transplanted Wnt1/iR1 tumors rapidly regress after inhibiting FGFR1 signaling following treatment with BGJ398. **a** Histological analysis of transplanted Wnt1/iR1 tumors as compared to spontaneous tumors. *Upper panel*: Hematoxylin and eosin (*H&E*) stain of spontaneous and transplanted Wnt1/iR1 tumors. *Lower panel*: Immunofluorescence double staining of luminal K8 (*green*) and basal K5 markers (*red*) in spontaneous and transplanted Wnt1/iR1 tumor sections. Nuclear staining is shown in *blue* (DAPI). **b** Immunofluorescence staining and quantification for vasculature using anti-CD31 in spontaneous and transplanted Wnt1/iR1 tumors; *p* = 0.51. **c** Immunofluorescence double staining for basal K5 (*green*) and HA epitope (*red*), and luminal K8 (*green*) and HA epitope (*red*) in spontaneous and transplanted Wnt1/iR1 tumor sections. Nuclear staining is shown in *blue* (DAPI) in all panels. **d** BGJ398 treatment scheme. **e** Tumor regression curve after receiving BGJ398 treatment, compared to control group treated with vehicle. Eleven mice in BGJ398 treatment group, four mice in vehicle treatment control group, 15 mice in total. **f** Immunofluorescence staining of the S phase proliferation marker BrdU, proliferative marker Ki67 and apoptotic marker cc3 in tumors from control and treatment groups (48 hours after BGJ398 treatment). **g** Quantification of immunofluorescence staining of nuclear BrdU, Ki67 and cc3 in tumor sections. BrdU (*n* ≥ 7): ****p < 0.0001; Ki67 (*n* ≥ 4): **p < 0.005; cc3 (*n* ≥ 6): **p < 0.005. Comparisons represent control versus 48 hours after BGJ398 treatment. Values are shown as mean ± SEM. *BrdU* Bromodeoxyuridine, *cc3* Cleaved caspase 3, *DAPI* 4′, 6-Diamidino-2-phenylindole

The bigenic MMTV-Wnt1/iR1 mammary tumor model has multiple advantages, such as an intact microenvironment, reproducible tumor kinetics and a consistent genetic background. Thus, we transplanted the tumor tissues from Wnt1/iR1 spontaneous tumors to the cleared fat pads of syngeneic FvB mice. Transplanted Wnt1/iR1 tumors maintained a similar histology as compared to spontaneous tumors (Fig. 2a). Tumors were stained for the luminal marker Keratin 8 (K8) and the basal marker Keratin 5 (K5). Transplanted Wnt1/iR1 tumors displayed a large expansion of the luminal compartment, which is consistent with the Wnt1/iR1 spontaneous tumors (Fig. 2a). Additionally, the vasculature appears similar in the transplanted as compared to the spontaneous tumors (Fig. 2b). Since the Wnt1/iR1 tumor cells express an epitope HA tagged iFGFR transgene, immunofluorescence staining for K8, K5 and the HA tag also were performed to verify if the tumors were derived from the original transplants (Fig. 2c). The overlapping expression of K8 and HA, but not K5 and HA, was observed in the transplanted tumors, as seen previously in spontaneous tumors [20]. These results indicate that the transplanted Wnt1/iR1 tumors maintained characteristics similar to spontaneous Wnt1/iR1 tumors, and can be used as a preclinical FGFR1-driven mouse mammary tumor model.

NVP-BGJ398 (BGJ398) is a potent and selective inhibitor of the FGFR family of receptor tyrosine kinases [22]. To assess the effect of BGJ398 on Wnt1/iR1 tumors, the Wnt1/iR1 transplanted tumors were consecutively treated with this inhibitor for 10 days (Fig. 2d) resulting in rapid tumor regression (Fig. 2e). Immunofluorescence staining of S phase proliferation marker BrdU, proliferation marker Ki67, and apoptotic marker cc3 showed a dramatic decrease in both cell proliferation and an increase in cell apoptosis 48 hours after BGJ398 treatment (Fig. 2f and g). In particular, increased apoptosis occurred mostly within the luminal cells, resulting in the shrinkage of the expanded luminal compartment (Additional file 2: Figure S1A). According to the earlier observations that FGFR1 signaling enhanced protein translation pathways [20], we performed immunoblot assay on cell lysates from the control and BGJ398-treated tumors. The results showed that the translation pathway components, p-mTOR and p-4EBP1, were markedly inhibited 24 hours after BGJ398 treatment (Additional file 2: Figure S1B). RPPA results further showed that key components of protein translation pathways, such as p70S6K, p-p70S6K, p-mTOR, and p-4E-BP1, were inhibited 6 and 24 hours after treatment (Additional file 3: Figure S1C). Downregulation of components of the MAPK pathway, a major downstream pathway of FGFR1, was also observed, such as p-MEK1, c-Fos, and p-c-Fos (Additional file 2: Figure S1C).

Taken together, these results confirmed that the transplanted tumor model exhibited a similar histological pattern to the spontaneous Wnt1/iR1 tumors. The transplanted tumors robustly responded to BGJ398 treatment, leading to a rapid regression as a result of the inhibition of both proliferation and an increase in apoptosis.

### After cessation of treatment, fully regressed Wnt/iR1 tumors recur spontaneously following a period of dormancy

**Fig. 3:**
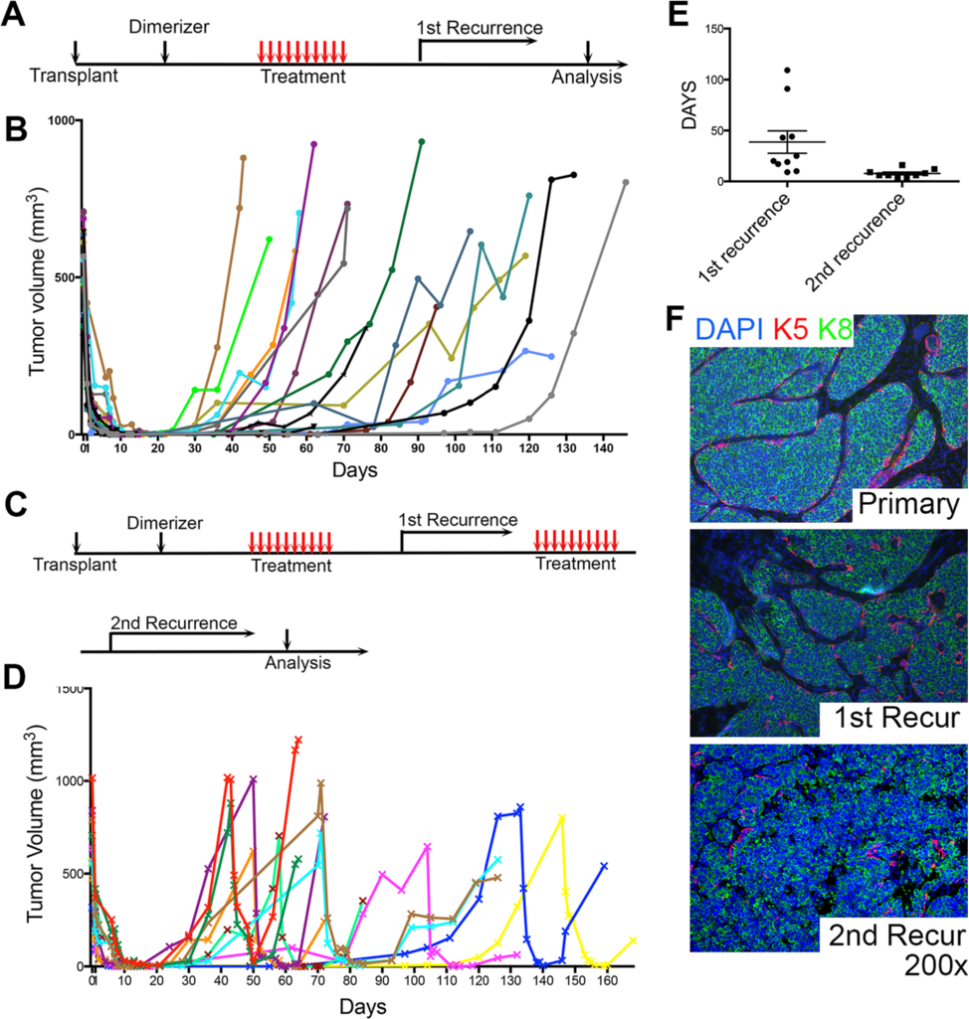
Fully regressed Wnt1/iR1 tumors after BGJ398 treatment recur slowly following cessation of treatment. **a** Scheme for BGJ398 treatment and tumor recurrence (first recurrence, *n* = 16). **b** Timing of tumor regression and recurrence for individual Wnt1/iR1 tumors. BGJ398 treatment was started on day 0, and stopped on day 10. **c** Scheme for BGJ398 treatment and tumor recurrence (second recurrence, *n* = 7). **d** Timing of tumor regression, first and second recurrence for individual Wnt1/iR1 tumors. **e** Comparison of the recurrent latency between the first and the second recurrence. Latency is calculated from the day of BGJ398 withdrawal to the day when the recurrent tumor reached 100 mm^3^; *p* < 0.05. **f** Immunofluorescence double staining of K5 (*red*) and K8 (*green*) in primary tumors and recurrent tumors. Nuclear staining is shown in *blue* (4′, 6-diamidino-2-phenylindole (*DAPI*)) in all panels

Downregulation of FGFR1 signaling in the established Wnt1/iR1 mammary tumors resulted in rapid regression to a nonpalpable state. However, all mice developed spontaneous tumor recurrences ranging from 1 to 4 months following the cessation of treatment (Fig. 3a and b). Regardless of the initial tumor size prior to treatment, tumors showed similar kinetics of regression as well as recurrence (Additional file 3: Figure S2A–C). Injection of dimerizer during the course of dormancy had no effect on promoting tumor recurrence (Additional file 3: Figure S2G and H). Furthermore, when BGJ398 treatment was extended from 10 days to 20 days, no significant difference in the latency of tumor recurrence was observed (Additional file 3: Figure S2D and E). In addition, no differences in change of body weight were detected in both treatment groups (Additional file 3: Figure S2F). However, three out of eight mice died during the extended 20-day treatment (0/5 dead in the control group). These data suggest that sustained BGJ398 treatment did not affect the kinetics of tumor regression or recurrence, and because of apparent toxicity was not warranted. Together, these results suggest that 10-day BGJ398 treatment is sufficient to kill the majority of tumor cells without reducing body weight, leaving a nonpalpable mass of malignant cells.

Most of the primary recurrent tumors (13 out of 16 mice) were still sensitive to treatment with BGJ398, but recurred when treatment was stopped (Fig. 3c and d). Interestingly, the second recurrence occurred with shorter latency (7.8 ± 1.4 days vs. 38.7 ± 11.0 days) (Fig. 3e). Half of the secondary recurrent tumors (three out of six mice) still responded to BGJ398 treatment but recurrence again occurred (Additional file 4: Figure S3A and B). The latency for the third recurrence was much shorter than the first and second recurrences (22.3 ± 8.7 days vs. 6.7 ± 3.1 days vs. 3.8 ± 1.8 days) (Additional file 4: Figure S3C). In addition, 18 % (3 out of 16) of primary recurrent tumors and 50 % (three out of six) secondary recurrent tumors eventually developed treatment resistance (Additional file 5: Figure S4A). To test if the recurrent tumors were derived from the primary tumors, immunofluorescence co-staining was performed for either the luminal marker K8 and the HA epitope or the basal marker K5 and the HA epitope (Additional file 6: Figure S5C and D). The recurrent tumors exclusively co-expressed K8 with the HA tag, indicating that these tumor cells were derived from the original transplanted Wnt1/iR1 tumor. Although the recurrent tumors still displayed extensive luminal compartments similar to the primary tumors, these luminal cells were more disorganized and expanded (Fig. 3f). In addition, the basal cell layer was further disrupted and often lost in the second recurrence (Fig. 3f). Moreover, immunofluorescence analysis revealed an increased expression of Ki67-positive cells as compared to the primary tumors (Additional file 6: Figure S5A and B). In summary, these data revealed that most recurrent tumors were still sensitive to BGJ398 treatment. However, they were not eliminated and subsequent recurrences appeared with altered histology, accompanied by a further loss of luminal organization, a loss of organization of the basal layer, and an increase in proliferating cells. This suggests that targeting FGFR1 alone was not sufficient to eradicate the residual Wnt1/iFGFR1 tumor cells.

### Residual tumor cells are surrounded by extensive stroma exhibiting a fibrotic response after BGJ398 treatment

**Fig. 4:**
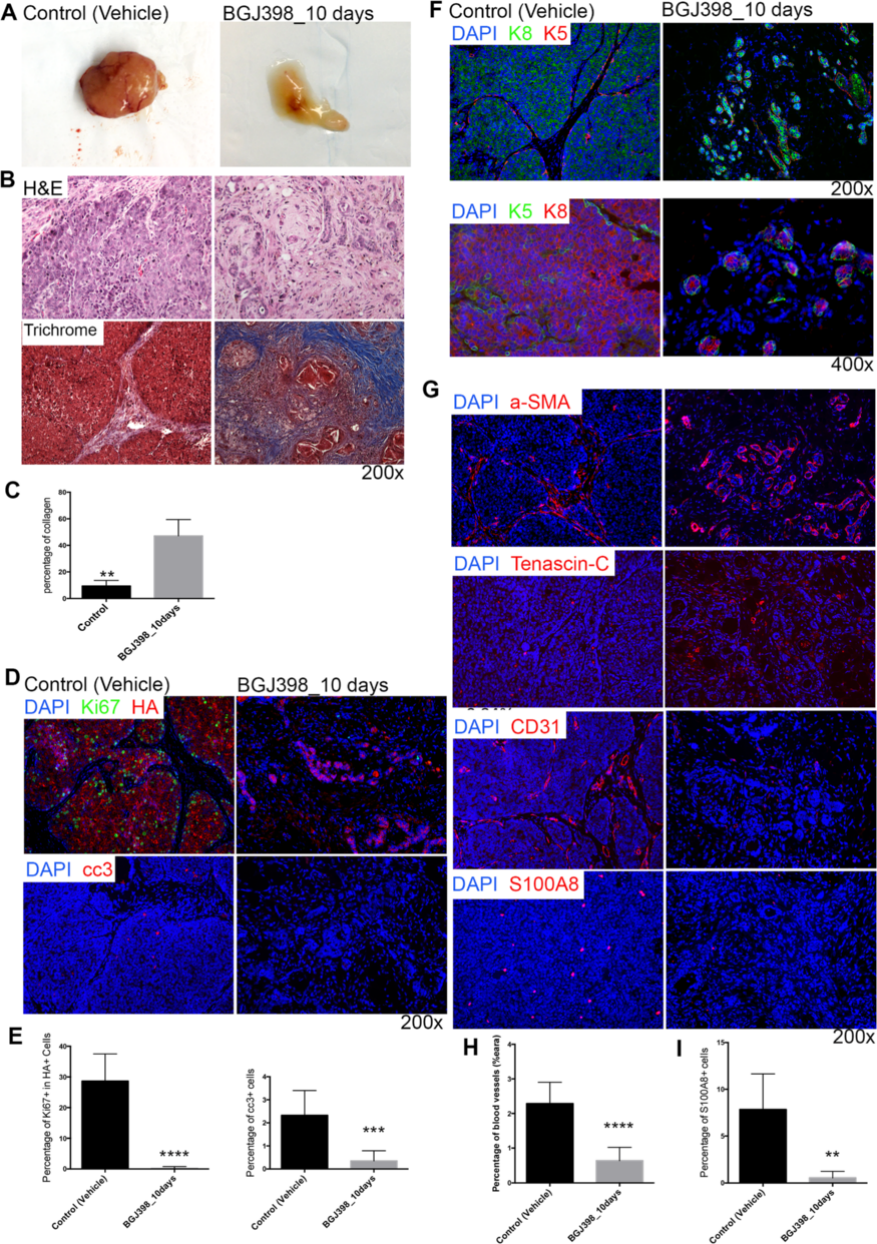
Residual tumor cells are surrounded by extensive stroma exhibiting a fibrotic response after BGJ398 treatment. **a** Mammary glands after 10-day BGJ398 treatment as compared to controls. **b** Histological analysis of tumors in BGJ398 treatment groups (10 days of treatment) as compared to control group treated with vehicle. *Upper panel*: Hematoxylin and eosin (*H&E*) staining. *Lower panel*: Trichrome staining. **c** Quantification of percentage of collagen in tumors. **p < 0.005, comparisons represent control versus 10 days after BGJ398 treatment. Values are shown as mean ± SEM. **d** Immunofluorescence double staining of proliferation marker Ki67 (*green*) in HA (*red*)-positive tumor cells and apoptosis marker cc3 (*red*) in tumor cells after 10 days BGJ398 treatment as compared to control group treated with vehicle. **e** Quantification of Ki67-positive cells in HA-positive cells after 10 days BGJ398 treatment as compared to a control group treated with vehicle alone, ****p < 0.0001; quantification of cc3-positive cells after 10 days BGJ398 treatment as compared to control group treated with vehicle alone, ***p = 0.0009. **f** Immunofluorescence double staining of K5 and K8 in tumors treated with BGJ398 for 10 days as compared to tumors in control group treated with vehicle. *Upper row*: K8 (*green*) and K5 (*red*), magnification 200×. *Lower row*: K8 (*red*) and K5 (*green*), magnification 400×. **g** Immunofluorescence staining of α-SMA, tenascin-C, CD31 and S100A8 in tumors treated with BGJ398 for 10 days as compared to tumors in control group treated with vehicle. **h** Quantification of vasculature after 10 days BGJ398 treatment as compared to control group treated with vehicle alone, ****p < 0.0001. **i** Quantification of S100A8-positive cells after 10 days BGJ398 treatment as compared to control group treated with vehicle alone, **p = 0.0012. *α-SMA* alpha smooth muscle actin, *cc3* Cleaved caspase 3, *DAPI* 4′, 6-Diamidino-2-phenylindole

Next, we analyzed the residual tumor cells in the fat pads after BGJ398 treatment. At the end of 10-day treatment, the tumors were no longer palpable (Fig. 4a). Hematoxylin and eosin staining staining showed that a few epithelial cells in the fat pads were left after the treatment, surrounded by a large amount of fibrous tissue (Fig. 4b). Trichrome staining showed that the dense stroma was enriched in collagen (Fig. 4b and c). However, expression of the proliferation marker Ki67 was not detected in the residual epithelial cells nor was the apoptosis marker cc3 (Fig. 4d and e), suggesting a state of dormancy [30]. Interestingly, double immunofluorescence staining for K8 and K5 shows that the dormant cells were organized into what appeared to be a normal mammary gland structure, with a basal cell layer and a luminal compartment (Fig. 4f).

To characterize the stroma around residual tumor cells, immunofluorescence staining was performed using antibodies against alpha smooth muscle actin (α-SMA^+^; myoepithelial cell marker), tenascin-C (tumor stromal marker), and also CD31 as a vasculature marker (Fig. 4g). In a comparison of MMTV-Wnt1 to bigenic MMTV-Wnt1/iFGFR1 tumors we observed a marked increase in anti-S100A8 staining (Additional file 7: Figure S6A), which could represent either neutrophils or myeloid-derived suppressor cells (MDSCs). Further characterization revealed a positive correlation of S100A8 staining with MDSCs by FACS analysis using anti-CD11b and anti-GR1 antibodies (Additional file 7: Figure S6B) as well as anti-Ly6G (data not shown). Residual tissue after 10 days treatment exhibited a more organized and continuous α-SMA^+^ cell layer which encapsulated the residual epithelial cells, in contrast to the untreated tumors which had a disrupted and sparse α-SMA^+^ layer (Fig. 4g). Moreover, there was increased expression of tenascin-C in the stroma. In addition, the vasculature was disrupted and infiltrating MDSCs were absent in the residual tissue after BGJ398 treatment (Fig. 4g–i). These findings suggest that BGJ398 treatment resulted in disrupted vasculature and markedly decreased tumor-infiltrating MDSCs. At the same time following treatment there was an increased expression of tenascin-C in the stroma, and the α-SMA^+^ myoepithelial cell layers were reconstituted around the dormant epithelial tumor cells, which displayed a normal mammary gland basal-luminal structure.

### Recurrent tumors contain the collagen-enriched stroma with upregulation of tenascin-C and phosphorylated EGFR expression

**Fig. 5:**
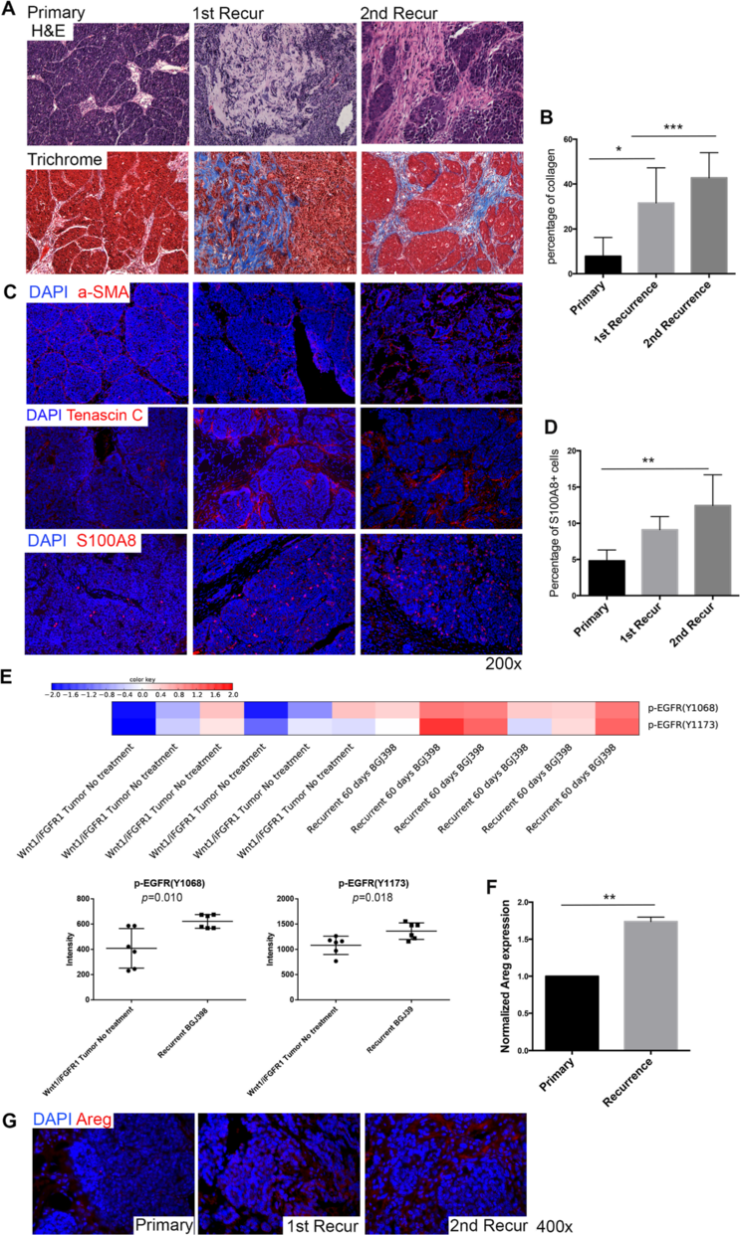
Collagen-enriched stroma in the recurrent tumors with increased tenascin-C and p-EGFR expression. **a** Hematoxylin and eosin (*H&E*) and trichrome staining of primary and recurrent tumors. **b** Quantification of percentage of collagen in primary, first recurrent, and second recurrent tumors. *p < 0.05, ***p < 0.001, comparisons represent primary versus first recurrence versus second recurrence. Values are shown as mean ± SEM. **c** Immunofluorescence staining of α-SMA, tenascin-C and S100A8 in primary and recurrent tumors. Nuclear staining is shown in *blue* (DAPI; and subsequent panels). **d** Quantification of S100A8+ MDSCs in recurrent tumors (*n* ≥ 5). **p < 0.005, comparisons represent primary versus first recurrence versus second recurrence. Values are shown as mean ± SEM. **e** p-EGFR expression levels in recurrent Wnt1/iR1 tumors through RPPA analysis. **f** Quantitative RT-PCR analysis of Areg normalized to GAPDH (*n* = 3). **p < 0.005. Values are shown as mean ± SEM. **g** Immunofluorescence staining of Areg in primary and recurrent tumors. *Areg* Amphiregulin, *α-SMA* alpha smooth muscle actin, *DAPI* 4′, 6-Diamidino-2-phenylindole, *FGFR1* Fibroblast growth factor receptor 1, *p-EGFR* Phosphorylated epidermal growth factor receptor

To investigate whether the stroma is enriched in the recurrent tumors, we collected the recurrent tumors and performed histological analysis. The recurrent tumors showed increased fibrosis similar to the stroma from the residual tissue (Fig. 5a). Trichrome staining further demonstrated that the stroma was also enriched in collagen (Fig. 5a and b). Immunofluorescence staining showed elevated expression of tenascin-C in the stroma as compared to the primary tumors (Fig. 5c). S100A8 staining also suggested that there was an increase in the number of MDSCs in the recurrent as compared to the primary tumors (Fig. 5c and d). However, no difference was detected in the expression pattern of α-SMA^+^ between recurrent and primary tumors (Fig. 5c). RPPA performed to investigate the possible molecular mechanisms involved in tumor recurrence indicated that the recurrent tumors had elevated phosphorylated EGFR (p-EGFR) expression (Fig. 5e). The upregulation of p-EGFR was also observed in the recurrent tumors by immunoblot assay (Additional file 8: Figure S7D). An increase of Areg, one of the principal EGFR ligands expressed in the developing mammary gland, was also detected in the recurrent as compared to the primary tumors (Fig. 5f and g). Together, these data revealed dense collagen fibrosis, increased expression of tenascin-C, increased infiltration of MDSCs in the stroma of the recurrent tumors, and upregulation of p-EGFR signaling as well as its ligand Areg in the recurrent tumors.

### Combinatorial blocking of EGFR and FGFR signaling significantly delays tumor recurrence, along with the loss of stroma in recurrent tumors

**Fig. 6:**
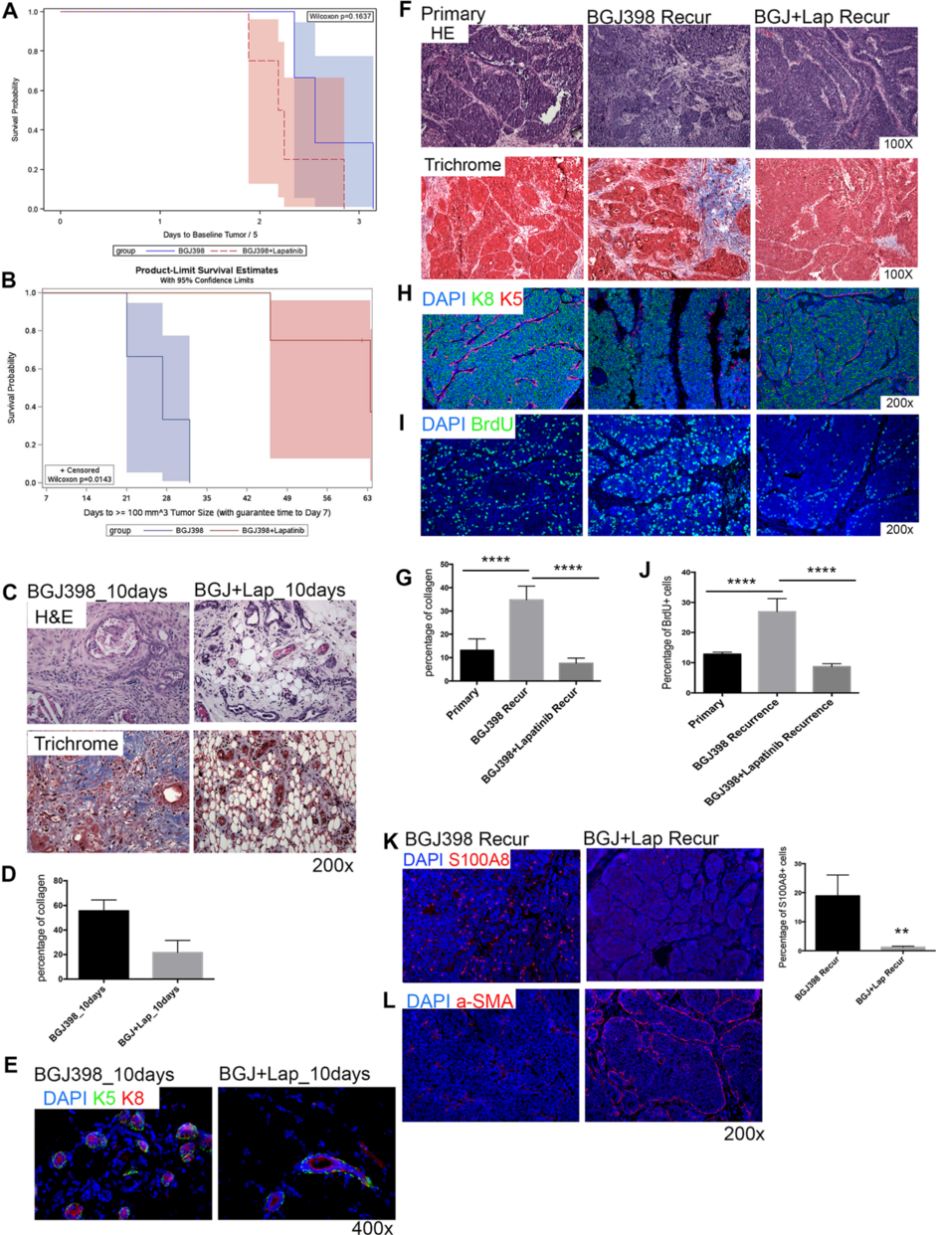
Inhibition of both EGFR and FGFR signaling significantly delays tumor recurrence. **a** Survival analysis of tumor regression after single BGJ398 treatment or combined BGJ398 and lapatinib treatment. Six mice in the single BGJ398 treatment group, eight mice in the combined BGJ398 and lapatinib treatment group, 14 mice in total; *p* = 0.5271. **b** Survival analysis of tumor recurrence after single BGJ398 treatment or combined BGJ398 and lapatinib treatment. Six mice in the single BGJ398 treatment group, eight mice in the combined BGJ398 and lapatinib treatment group, 14 mice in total; *p* = 0.0005. **c** Histological analysis of tumors treated with BGJ398 alone or combined BGJ398 and lapatnib for 10 days. *Upper panel*: Hematoxylin and eosin (*H&E*) staining. *Lower panel*: trichrome staining. **d** Quantification of percentage of collagen in tumors treated with BGJ398 alone or combined BGJ398 and lapatnib for 10 days; *p* = 0.0079. **e** Immunofluorescence double staining of K5 (*green*) and K8 (*red*) in tumors treated with BGJ398 alone or combined BGJ398 and lapatnib for 10 days. Nuclear staining is shown in *blue* (DAPI). **f** Histological analysis of primary tumors and tumors after single treatment or combined treatment. *Upper panel*: H&E staining. *Lower panel*: trichrome staining. **g** Quantification of percentage of collagen in primary tumors, recurrent tumors from BGJ398 treatment, and recurrent tumors from BGJ398 and lapatinib treatment; ****p < 0.0001. Values are shown as mean ± SEM. **h**. Immunofluorescence double staining of K5 (*red*) and K8 (*green*) in primary tumors and tumors after single treatment or combined treatment. Nuclear staining is shown in *blue* (DAPI; and subsequent panels). **i** Immunofluorescence staining of BrdU in primary tumors and tumors after single treatment or combined treatment. **j** Quantification of BrdU-positive cells in recurrent tumors from combined treatment group (n = 5); ****p < 0.0001. Values are shown as mean ± SEM. **k** Immunofluorescence staining and quantification of S100A8 (*red*) in tumors after single treatment or combined treatment; *p* = 0.0084. **l** Immunofluorescence staining of α-SMA (*red*) in tumors after single treatment or combined treatment. *α-SMA* alpha smooth muscle actin, *BGJ* BGJ398, *BrdU* Bromodeoxyuridine, *DAPI* 4′, 6-Diamidino-2-phenylindole, *Lap* Lapatinib, *Recur* Recurrence

To test if the EGFR pathway is necessary for recurrence, we utilized an EGFR inhibitor, lapatinib, to inhibit EGFR pathways in vivo. Although lapatinib also inhibits ErbB2, our RPPA results revealed no evidence for ErbB2 signaling in the primary and recurrent tumors (data not shown). Moreover, p-ErbB2 expression was not detectable in primary or recurrent tumors in this mouse model (Additional file 8: Figure S7D). The results also showed that lapatinib successfully inhibited p-EGFR expression (Additional file 8: Figure S7E). Mice treated with lapatinib alone showed no tumor regression as compared to control mice. Mice treated with BGJ398 plus lapatinib showed the same kinetics of tumor regression as compared to the BGJ398 group alone (Fig. 6a; Additional file 9: Figure S8). However, mice treated with both BGJ398 and lapatinib exhibited delayed tumor recurrence as compared to mice treated with single treatment of BGJ398 (Fig. 6b; Additional file 9: Figure S8). In order to investigate the mechanism that led to the delayed recurrence, we collected the residual tissue after treatment as well as recurrent tumors from both groups and performed histological analysis. We found that the residual tissue after combinatorial treatment showed less stroma but more adipocytes (Fig. 6c and d). The residual tumor cells in both treatment groups were organized into a normal mammary gland structure, with a basal cell layer and a luminal compartment (Fig. 6e). The recurrent tumors from the combinatorial treatment exhibited reduced stroma and collagen expression similar to that observed in the primary tumors (Fig. 6f and g). Unlike recurrent tumors in the BGJ398 treatment group, recurrent tumors from the combined treatment group showed organized expanded luminal cells surrounded by a defined basal cell layer (Fig. 6h). There were fewer proliferating cells in the recurrent tumors after the combined treatment (Fig. 6i and j). Interestingly, in contrast to the recurrent tumors that arose following the single treatment of BGJ398, which exhibited a large number of MDSCs, only a few MDSCs were observed in the recurrent tumors from the combined treatment group (Fig. 6k). In addition, increasing numbers of α-SMA^+^ myoepithelial cells were found in the recurrent tumors from the combined treatment group, and these myoepithelial cells formed continuous layers around the tumor epithelial cells (Fig. 6l). In addition, tumors treated with lapatinib for only 8 days contained reduced stroma as compared to tumors in the control group (Additional file 8: Figure S7A–C).

In summary, these results show that blocking both the FGFR1 and EGFR signaling pathways significantly delays tumor recurrence. Recurrent tumors from the combined treatment contained reduced stroma and MDSCs, and exhibited an increased myoepithelial cell organization.

## Discussion

In this study, a transplantable mouse model for Wnt1/iFGFR1-driven breast cancer was employed to demonstrate changes in the stromal microenvironment and EGFR signaling occurring during tumor dormancy and recurrence. Targeting FGFR1 alone resulted in rapid tumor regression, but tumors eventually recurred with latencies of 1 to 4 months. The tumors recurred from dormant residual cells surrounded by a large amount of collagen-enriched stroma. In addition, upregulation of tenascin-C and p-EGFR was observed in the recurrent tumors and inhibiting p-EGFR signaling significantly delayed recurrence without affecting initial tumor regression. These results provide insights in developing new strategies for cancer treatment.

Upon treatment with BGJ398, Wnt1/iR1 tumor cells stopped proliferating. A large number of cells immediately underwent apoptosis, and tumors shrank to a nonpalpable size within 5 days. This fits the oncogene addiction paradigm, which dictates that the survival of a cancer cell becomes unusually dependent on the activity of a single gene product [31]. Therefore, FGFR signaling might be a potentially promising therapeutic target in cancers exhibiting FGFR1 overexpression. However, tumors recurred after cessation of the BGJ398 treatment. Residual tumor cells remaining after treatment with BGJ398 led to subsequent recurrence. Moreover, although the initial recurrence was usually BGJ398-sensitive after several cycles of treatment, some of the recurrent tumors became invasive and developed resistance to BGJ398 treatment (Additional file 5: Figure S4).

In the present study, the residual dormant cells were surrounded by a collagen-enriched fibrotic stroma with upregulation of tenascin-C. The vasculature was disrupted, and tumor-infiltrating MDSCs were diminished at the end of the treatment. Increased α-SMA^+^ cells were observed around the residual epithelial cells. In the mammary gland, α-SMA^+^ myoepithelial cells act as a natural barrier and reside between the epithelial cells and the surrounding stroma. Molecules from the stroma must pass through the myoepithelial cell layer to reach the epithelial cells surrounding the lumen, and vice versa. Disruption of this structure causes alterations in microenvironment and is a prerequisite for ductal carcinoma in situ progression and breast tumor invasion [32]. These changes may have inhibited the ability of BGJ398 to target the residual cancer cells, thus rendering the residual cells resistant to the treatment. Another possibility is that BGJ398 treatment selected for a resistant subpopulation within the tumor—namely, the tumor-initiating cells, or “cancer stem cells (CSCs)”. The tumor dormancy and CSC theories have many similarities. For example, the dormancy theory suggests that there is a small subpopulation of dormant cells responsible for resistance and tumor recurrence. Likewise, the CSC hypothesis similarly predicts that a subset of self-renewing cancer cells is responsible for tumor initiation, therapy resistance, delayed cancer recurrence and metastatic progression. In addition, several biological mechanisms, such as cell cycle modifications, alteration of angiogenic processes, and modulation of antitumor immune responses, involved in controlling the dormant state of a tumor, may also be observed in CSCs. In fact, quiescence and immune escape are emerging hallmark features of at least some CSCs, suggesting that there is a significant overlap between dormant cancer populations and CSCs [33]. Unfortunately, with the current transplanted tumor model it was not feasible to isolate the small number of residual cells to directly test these hypotheses and a specific biomarker for FGFR signaling is not available. Future studies using a fluorescently and bioluminescently tagged model may help facilitate these studies.

The increasing collagen-enriched stroma observed in the residual tissue was also found in the recurrent tumors. Other studies have shown that fibrotic foci with dense collagen are often observed in primary tumors of breast cancer patients with lymph node metastases at high risk of recurrence [34, 35]. Recent evidence also shows that breast cancer cells in a dense collagen stromal environment are invasive, but dormant. These cells encapsulated in collagen can resist local and adjuvant therapies, leading to local recurrence [36]. A high level of procollagen type I, a marker for collagen synthesis, has been detected in the serum of patients with recurrent breast cancer [37]. A type I collagen-enriched fibrotic environment has been shown to be able to switch dormancy to proliferation and induce metastatic growth from these tumor cells [38]. More interestingly, we found that recurrent tumors show a loss of basal cell layers, resulting in direct contact between luminal cells and stromal cells. One of the key characteristics of invasive cancer progression is the loss of myoepithelial cell layer and basement membrane, since normal myoepithelial cells form a natural barrier against cancer cell invasion [39]. MDSCs are a heterogeneous population of immature myeloid cells, which may inhibit the immunosuppressive activity of T cells. The level of MDSCs also is correlated with disease burden and is a potential prognostic marker for breast cancer [40]. Our data revealed increased MDSC infiltration in the recurrent tumors, indicating that the recurrent tumor cells could be potentially invasive and metastatic, an observation that warrants further study.

**Fig. 7:**
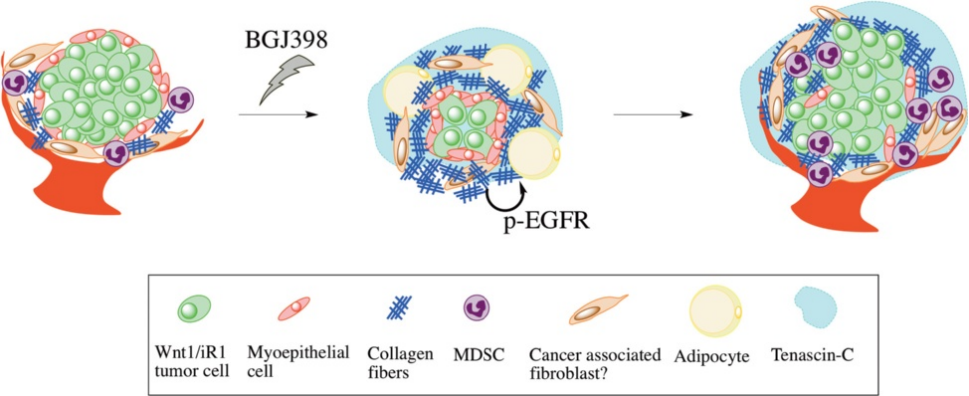
Model of mechanism of tumor recurrence after BGJ398 treatment. Before the treatment with the FGFR inhibitor, BGJ398, Wnt1/iR1 tumor cells are surrounded by α-SMA^+^ myoepithelial cells and stroma, which is comprised of stromal fibroblasts, MDSCs, blood vessels and collagen. Upon BGJ398 treatment, the majority of the tumor cells die, leaving a nonpalpable mass of tumor cells with remodeled stroma, including thickened myoepithelial cell layers, increased collagen, and upregulation of tenascin-C. Vasculature and MDSCs are eliminated after BGJ398 treatment. Over time, EGFR signaling is upregulated, accompanied by remodeled stroma and tumor recurrence. The stroma in the recurrent tumors maintains elevated tenascin-C and increased collagen expression. However, the recurrent tumors have a disrupted myoepithelial cell layer and increased MDSC infiltration indicating that they may be more invasive as compared to the primary tumors. *iR1* Inducible fibroblast growth factor receptor 1, *MDSC* Myeloid-derived tumor suppressor cell, *p-EGFR* Phosphorylated epidermal growth factor receptor

Tenascin-C, an extracellular matrix glycoprotein, is required for the active remodeling process of tissues, such as wound healing and inflammation [41]. Increased expression of tenascin-C has been also found in the stroma of various cancers and its expression is predictive of local recurrence, metastatic dissemination of cancer cells and anti-cancer treatment responsiveness in breast cancer [41]. Our results show that tenascin-C is upregulated upon the BGJ398 treatment, and high expression of tenascin-C is maintained during dormancy and recurrence. Because tenascin-C has EGF-like repeats which bind to EGFR with low affinity, it has been suggested to be an EGFR ligand originating from the extracellular matrix [42]. These data suggest that overexpression of tenascin-C in the recurrent tumors could lead to the activation of EGFR signaling. The RPPA data consistently showed upregulated p-EGFR in the recurrent tumors. Areg is another important EGFR ligand, especially in mammary gland development, and expression of Areg was increased in the recurrent tumors. EGFR signaling in the stroma is thought to be mediated by Areg in the developing mammary gland [43]. Previous studies using the iFGFR model have shown that Areg expression can be induced by FGFR1 and leads to activation of EGFR signaling to promote mammary tumorigenesis [44]. This and other studies suggest the existence of crosstalk between EGFR and FGFR signaling during mammary tumorigenesis. In vitro and in vivo data showed that combinatorial inhibition of the EGFR and FGFR signaling pathways in various human cancers results in better outcomes as compared to the single regimens alone [45, 46]. The current study, for the first time, showed that blocking EGFR signaling significantly delayed the recurrence of BGJ398-treated mammary tumors. Together, these findings suggest that the activation of EGFR signaling is correlated with stroma remodeling in tumor recurrence (Fig. 7), and that inhibition of EGFR signaling may help delay recurrence.

Although inhibiting both FGFR1 and EGFR resulted in delayed recurrence, no difference was observed in initial tumor regression as compared to inhibiting FGFR1 alone. This observation suggests that pathological complete response, defined as the absence of invasive cancer in breast and lymph nodes, following chemotherapy may not be the best index of neoadjuvant efficacy. Some chemotherapy regimes might have different disease outcomes, although they result in the same pathological complete response rates as compared to other regimes [47, 48]. Our results also provide additional support for the role of the stromal microenvironment in tumor dormancy and recurrence. In addition, the importance of EGFR signaling in tumor dormancy and recurrence of FGFR1-driven mammary tumors provides a further rationale for combinatorial treatments in breast cancers harboring FGFR1 amplification.

## Conclusion

Although breast cancer patient survival has been improved through early detection and adjuvant targeted therapies, many patients, though exhibiting no detectable minimal residual disease in some cases for many years, suffer relapse and tumor recurrence. Understanding the mechanisms involved in dormancy and tumor recurrence is important for developing new therapeutic strategies for cancer treatment. Our findings showed that stromal remodeling, accompanied by upregulation of EGFR signaling, is correlated with mammary tumor recurrence, providing new insights into the development of targeted therapies. The observation that combinatorial treatment resulted in delayed recurrence, but no difference in primary tumor regression, suggests that there also is a need for additional markers to evaluate neoadjuvant therapy efficacy besides pathologic complete response.

## Additional files

Additional file 1: Table S1. List of antibodies and reagents. (PDF 33 kb) Additional file 2: Figure S1. BGJ398 treatment in Wnt1/iR1 tumors results in rapid apoptosis of luminal cells and downregulation of protein translation pathways. A. Immunofluorescence double staining of K5 (*red*) and K8 (*green*) in tumors from control or treatment groups (48 hours after BGJ398 treatment). *Yellow arrows* indicate the areas of apoptosis. Nuclear staining is shown in *blue* (DAPI). B. Immunoblot analysis of p-mTOR and p-4E BP1 in Wnt1/iR1 tumors with BGJ398 treatment. Beta-actin was used as a loading control. C. Protein expression levels in Wnt1/iR1 tumors 6 and 24 hours after treated with BGJ398 as compared to control determined through RPPA analysis. (TIF 18070 kb) Additional file 3: Figure S2. Initial tumor size, extended BGJ398 treatment, and dimerizer injection have no significantly effect on tumor recurrence. A. Timing of tumor regression and recurrence for individual Wnt1/iR1 tumors from two groups of tumors with different starting sizes. B. Analysis of starting sizes of Wnt1/iR1 tumors for BGJ398 treatment (*n* = 3); ***p* < 0.01. Values are shown as mean ± SEM. C. Analysis of recurrent latency between two groups with different starting sizes (*n* = 3); *p* = 0.78. D. Timing of tumor regression and recurrence for individual Wnt1/iR1 tumors from two groups of tumors with different treatment regimens (10 vs. 20 days). E. Analysis of recurrence latency (*n* = 5); *p* = 0.89. F. Analysis of body weight loss. G. Timing of tumor regression and recurrence for individual Wnt1/iR1 tumors receiving dimerizer injection during dormancy or not. H. Analysis of recurrence latency (*n* = 3); *p* = 0.6. (TIF 22648 kb) Additional file 4: Figure S3. Tumors recur much faster in the third recurrence as compared to the first and second recurrences. A. Scheme for BGJ398 treatment and tumor recurrence (third recurrence). B. Timing of tumor regression and recurrence for individual Wnt1/iR1 tumors. C. Comparison of the recurrence latency between the first, the second and the third recurrences. Latency is calculated from the day of BGJ398 withdrawal to the day when the recurrent tumors reached 100 mm^3^ (*n* = 3); ***p* < 0.001. (TIF 21045 kb) Additional file 5: Figure S4. Recurrent tumors eventually develop resistance and become more invasive. A. Timing of tumor resistance for individual Wnt1/iR1 tumors. *Red arrows* indicate when resistance occurred during treatment. B. Immunofluorescence double staining of K5 (*red*) and K8 (*green*) in primary, first and second recurrent and resistant tumors. (TIF 1845 kb) Additional file 6: Figure S5. Recurrent tumors are derived from the original transplanted tumor cells and have increased proliferation. A. Immunofluorescence staining of Ki67 in primary, first and second recurrent tumors. Nuclear staining is shown in *blue* (DAPI). B. Quantification of Ki67-positive cells in primary, first recurrent and second recurrent tumors. (*n* = 4); ***p* < 0.01. Values are shown as mean ± SEM. C. Immunofluorescence double staining of K5 (*green*) and HA (*red*) in recurrent tumors. Nuclear staining is shown in *blue* (DAPI) in all panels. D. Immunofluorescence double staining of K8 (*green*) and HA (*red*) in recurrent tumors. Nuclear staining is shown in *blue* (DAPI) in all panels. (TIF 2877 kb) Additional file 7: Figure S6. The number of S100A8-positive cells is positively correlated with the number of Gr1^+^CD11b^high^ cells. A. Immunofluorescence staining of S100A8 in Wnt1 and Wnt1/iR1 tumors. Nuclear staining is shown in *blue* (DAPI). B. FACS analysis of peripheral blood Gr1 + CD11bhigh subpopulation in control (no tumor), Wnt1 tumor and Wnt1/iR1 tumor mouse models. (TIF 14790 kb) Additional file 8: Figure S7. Lapatinib treatment reduces collagen expression in the tumor stroma. A. H&E of tumors from control or treatment groups with 8 days of lapatinib treatment. B. Trichrome staining of tumors from control or treatment groups with 8 days of lapatinib treatment. C. Quantification of percentage of collagen in tumors from control and 8-day lapatinib treatment group; ****p* < 0.001. Values are shown as mean ± SEM. D. Immunoblot analysis of p-ErbB2 in primary Wnt1/iR1 tumors, recurrent tumors arising from BGJ398 treatment or BGJ398 + lapatinib treatment, and tumors treated with lapatinib for 8 days. SKBR3 cell lysate was used as a positive control for p-ErbB2. Beta-actin was used as a loading control. E. Immunoblot analysis of p-EGFR (Y1068) in Wnt1/iR1 tumors treated with lapatinib for 8 days, as well as recurrent tumors arising from BGJ398 treatment or BGJ398 + lapatinib treatment, as compared to control group treated with vehicle. Beta-actin was used as a loading control. (TIF 32982 kb) Additional file 9: Figure S8. Individual plots of tumor regression and recurrence in the BGJ398 and BGJ398 and lapatinib treatment groups. Six mice in the single BGJ398 treatment group, eight mice in the combined BGJ398 and lapatinib treatment group, 14 mice in total. (TIF 13485 kb)

## Abbreviations

Areg: Amphiregulin
α-SMA: Alpha smooth muscle actin
BrdU: Bromodeoxyuridine
cc3: Cleaved caspase 3
CSC: Cancer stem cell
DAPI: 4′, 6-Diamidino-2-phenylindole
DMSO: Dimethyl sulfoxide
EGFR: Epidermal growth factor receptor
FGFR: Fibroblast growth factor receptor
iFGFR1: Inducible fibroblast growth factor receptor 1
iR1: Inducible FGFR1
MDSC: Myeloid-derived tumor suppressor cell
PCR: Polymerase chain reaction
p-EGFR: Phosphorylated epidermal growth factor receptor
p.o.: Per oral
RPPA: Reverse phase protein array
Wnt1: Wingless-Type MMTV Integration Site Family, Member 1
