# Supplementary material for: Upregulation of EGFR signaling is correlated with tumor stroma remodeling and tumor recurrence in FGFR1-driven breast cancer
Source: Breast Cancer Res. 2015 Nov 18;17:141. doi: 10.1186/s13058-015-0649-1 (PMC4652386; doi:10.1186/s13058-015-0649-1)
Supplement: Additional file 1: Table S1. — List of antibodies and reagents. (PDF 33 kb) [file 13058_2015_649_MOESM1_ESM.pdf]

Supplementary Table 1: List of Antibodies and Reagents

Antibody/Reagents	Application	Manufacturer	Catalog#
BrdU-488	IF	Invitrogen	B35130
p-Histone H3	IF	Upstate	06-570
Keratin 5	IF	Covance	PRB-160P
Keratin 8	IF	Development Studies Hybridoma Bank	TROMA-I
HA	IF	Cell Signaling	2367
Ki67	IF	Vector Labs	VP-K451
cc3	IF	Cell Signaling	9661
S100A8	IF	R&D System	MAB3059
SMA- α	IF	Sigma	A2547
Tenascin-C	IF	Millipore	AB19013
CD31	IF	abcam	ab28364
Areg	IF	Santa Cruz	sc-74501
p-mTOR	WB	Cell Signaling	2974
p-4EBP1	WB	Cell Signaling	2855
β-actin	WB	Abcam	ab8227
p-EGFR (Y1068)	WB	Cell Signaling	3777
p-HER2	WB	Cell Signaling	6942
GR1	FACS	BioLegend	108407
CD11b	FACS	abcam	ab8878
Phosphatease	WB	Cell Signaling	5872
Protease inhibitors	WB	Roche	4693116001
