# Supplementary figures and images for: Upregulation of EGFR signaling is correlated with tumor stroma remodeling and tumor recurrence in FGFR1-driven breast cancer
Source: Breast Cancer Res. 2015 Nov 18;17:141. doi: 10.1186/s13058-015-0649-1 (PMC4652386; doi:10.1186/s13058-015-0649-1)

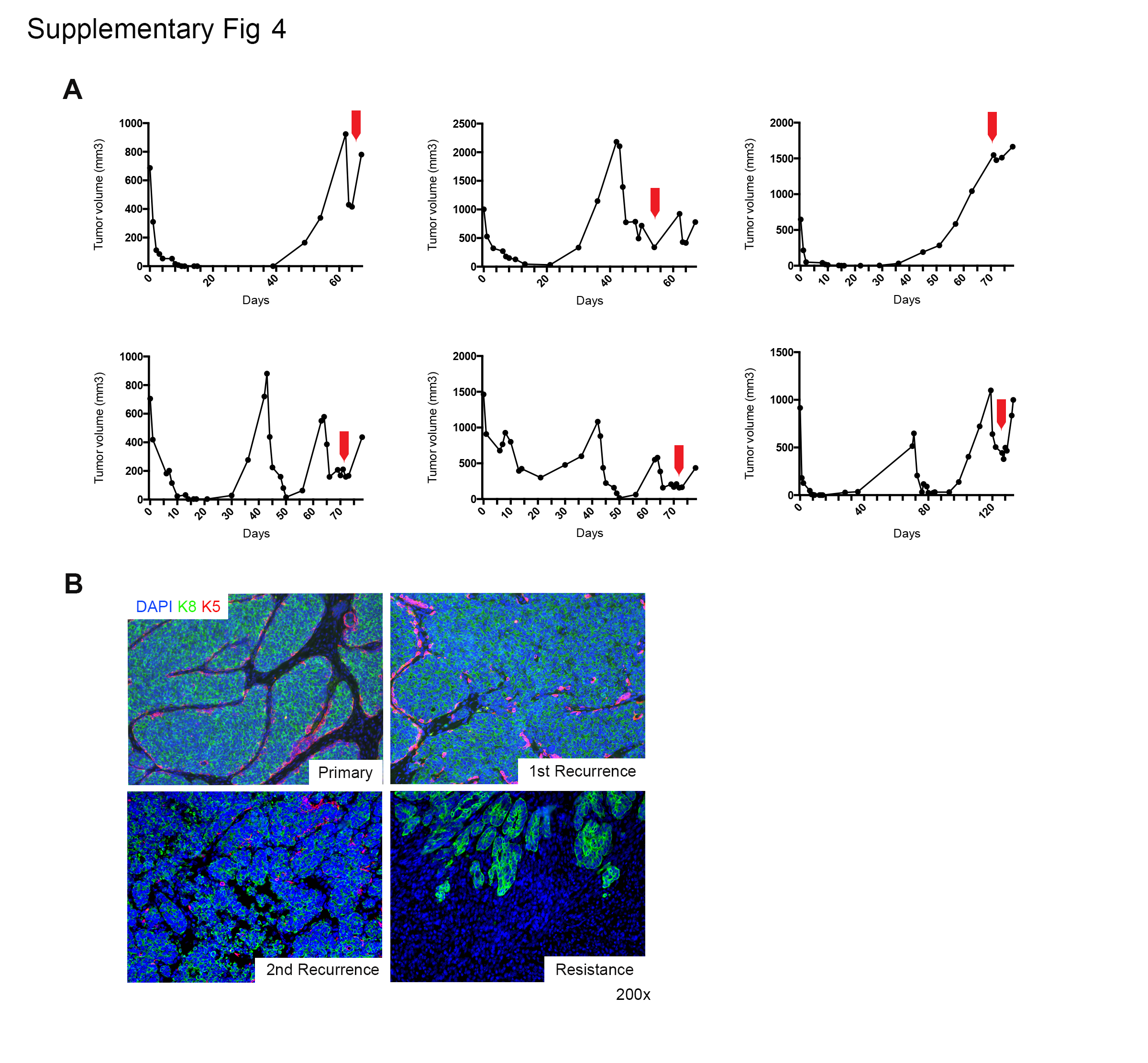

Supplement: Additional file 2: Figure S1. — BGJ398 treatment in Wnt1/iR1 tumors results in rapid apoptosis of luminal cells and downregulation of protein translation pathways. A. Immunofluorescence double staining of K5 (red) and K8 (green) in tumors from control or treatment groups (48 hours after BGJ398 treatment). Yellow arrows indicate the areas of apoptosis. Nuclear staining is shown in blue (DAPI). B. Immunoblot analysis of p-mTOR and p-4E BP1 in Wnt1/iR1 tumors with BGJ398 treatment. Beta-actin was used as a loading control. C. Protein expression levels in Wnt1/iR1 tumors 6 and 24 hours after treated with BGJ398 as compared to control determined through RPPA analysis. (TIF 18070 kb) [file 13058_2015_649_MOESM2_ESM.tif]

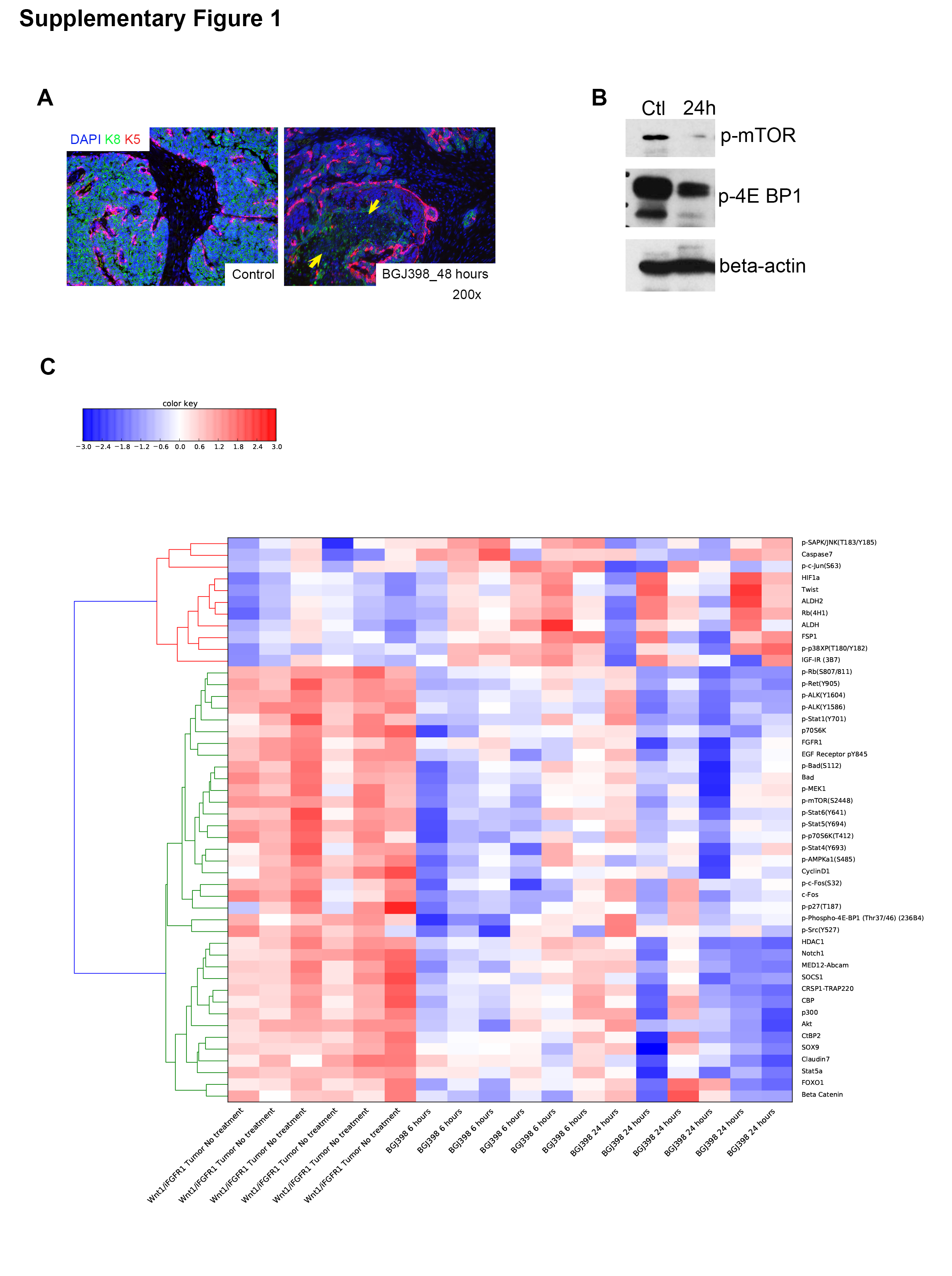

Supplement: Additional file 3: Figure S2. — Initial tumor size, extended BGJ398 treatment, and dimerizer injection have no significantly effect on tumor recurrence. A. Timing of tumor regression and recurrence for individual Wnt1/iR1 tumors from two groups of tumors with different starting sizes. B. Analysis of starting sizes of Wnt1/iR1 tumors for BGJ398 treatment (n = 3); **p < 0.01. Values are shown as mean ± SEM. C. Analysis of recurrent latency between two groups with different starting sizes (n = 3); p = 0.78. D. Timing of tumor regression and recurrence for individual Wnt1/iR1 tumors from two groups of tumors with different treatment regimens (10 vs. 20 days). E. Analysis of recurrence latency (n = 5); p = 0.89. F. Analysis of body weight loss. G. Timing of tumor regression and recurrence for individual Wnt1/iR1 tumors receiving dimerizer injection during dormancy or not. H. Analysis of recurrence latency (n = 3); p = 0.6. (TIF 22648 kb) [file 13058_2015_649_MOESM3_ESM.tif]

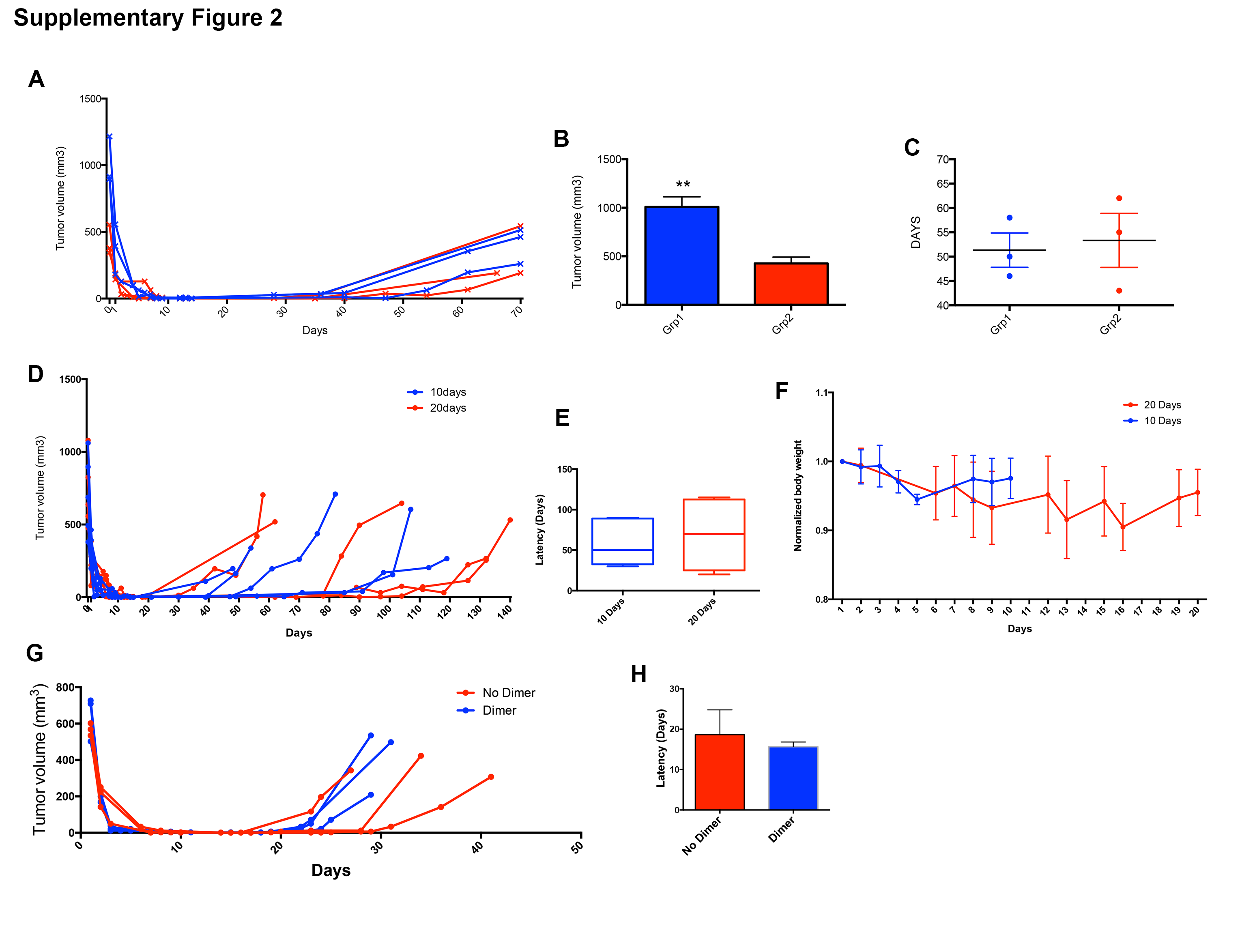

Supplement: Additional file 4: Figure S3. — Tumors recur much faster in the third recurrence as compared to the first and second recurrences. A. Scheme for BGJ398 treatment and tumor recurrence (third recurrence). B. Timing of tumor regression and recurrence for individual Wnt1/iR1 tumors. C. Comparison of the recurrence latency between the first, the second and the third recurrences. Latency is calculated from the day of BGJ398 withdrawal to the day when the recurrent tumors reached 100 mm3 (n = 3); **p < 0.001. (TIF 21045 kb) [file 13058_2015_649_MOESM4_ESM.tif]

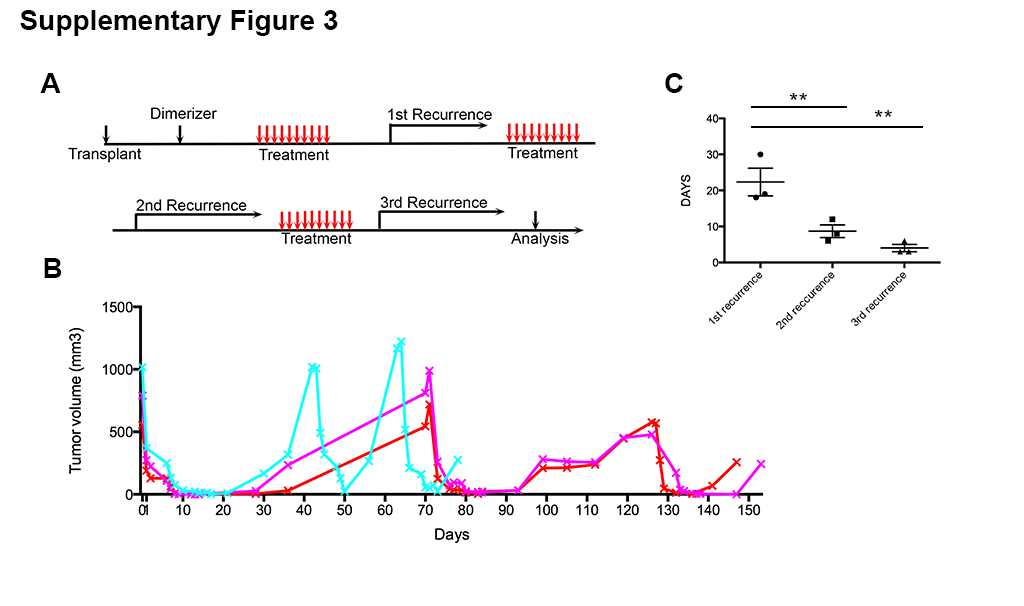

Supplement: Additional file 5: Figure S4. — Recurrent tumors eventually develop resistance and become more invasive. A. Timing of tumor resistance for individual Wnt1/iR1 tumors. Red arrows indicate when resistance occurred during treatment. B. Immunofluorescence double staining of K5 (red) and K8 (green) in primary, first and second recurrent and resistant tumors. (TIF 1845 kb) [file 13058_2015_649_MOESM5_ESM.tif]

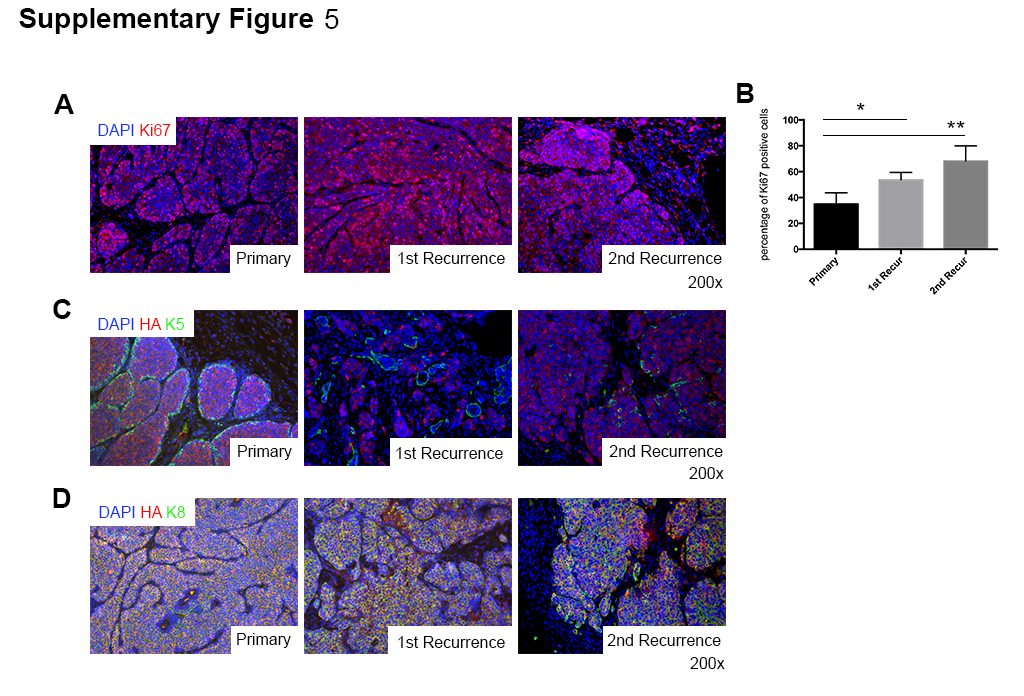

Supplement: Additional file 6: Figure S5. — Recurrent tumors are derived from the original transplanted tumor cells and have increased proliferation. A. Immunofluorescence staining of Ki67 in primary, first and second recurrent tumors. Nuclear staining is shown in blue (DAPI). B. Quantification of Ki67-positive cells in primary, first recurrent and second recurrent tumors. (n = 4); **p < 0.01. Values are shown as mean ± SEM. C. Immunofluorescence double staining of K5 (green) and HA (red) in recurrent tumors. Nuclear staining is shown in blue (DAPI) in all panels. D. Immunofluorescence double staining of K8 (green) and HA (red) in recurrent tumors. Nuclear staining is shown in blue (DAPI) in all panels. (TIF 2877 kb) [file 13058_2015_649_MOESM6_ESM.tif]

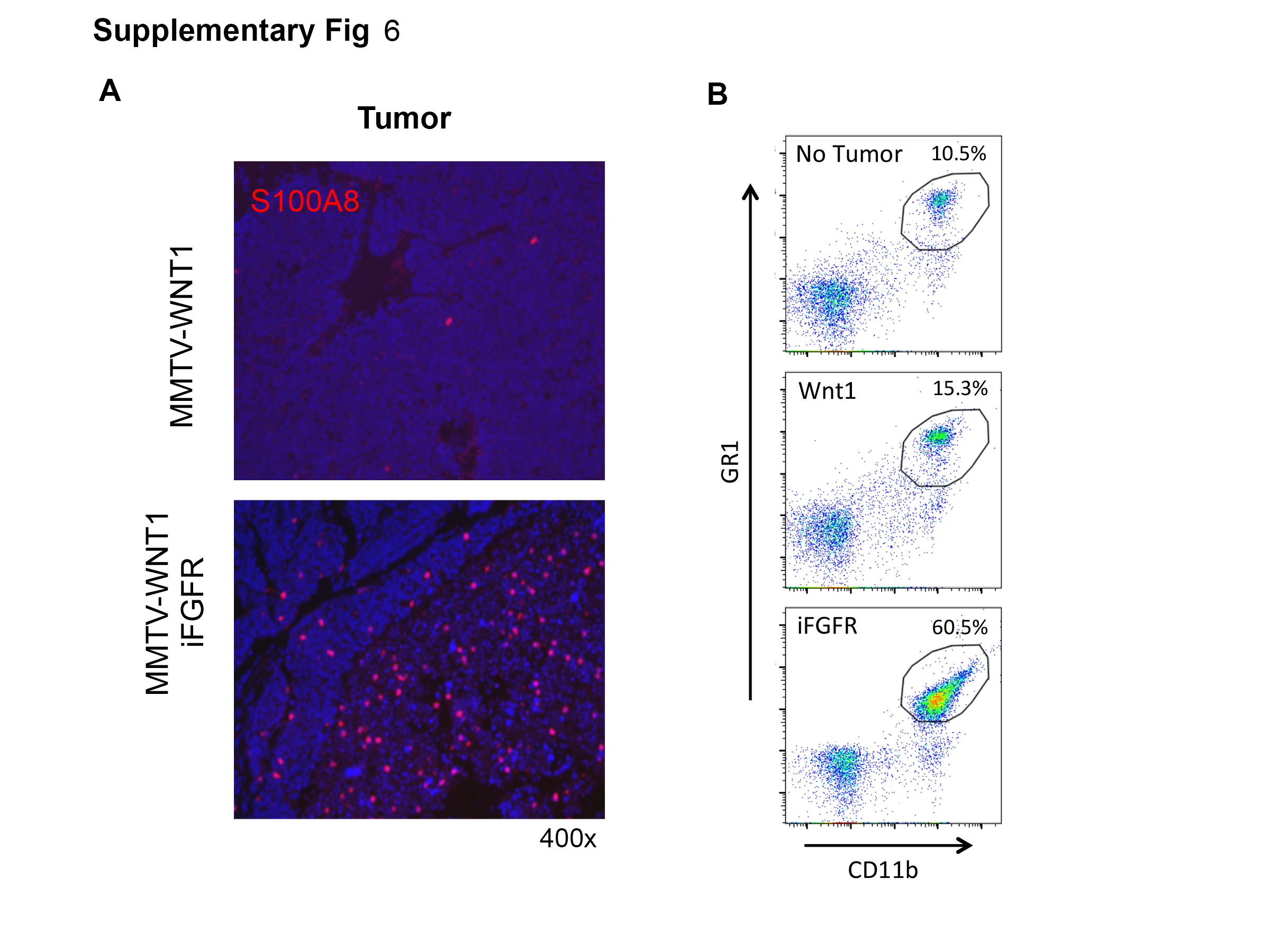

Supplement: Additional file 7: Figure S6. — The number of S100A8-positive cells is positively correlated with the number of Gr1+CD11bhigh cells. A. Immunofluorescence staining of S100A8 in Wnt1 and Wnt1/iR1 tumors. Nuclear staining is shown in blue (DAPI). B. FACS analysis of peripheral blood Gr1 + CD11bhigh subpopulation in control (no tumor), Wnt1 tumor and Wnt1/iR1 tumor mouse models. (TIF 14790 kb) [file 13058_2015_649_MOESM7_ESM.tif]

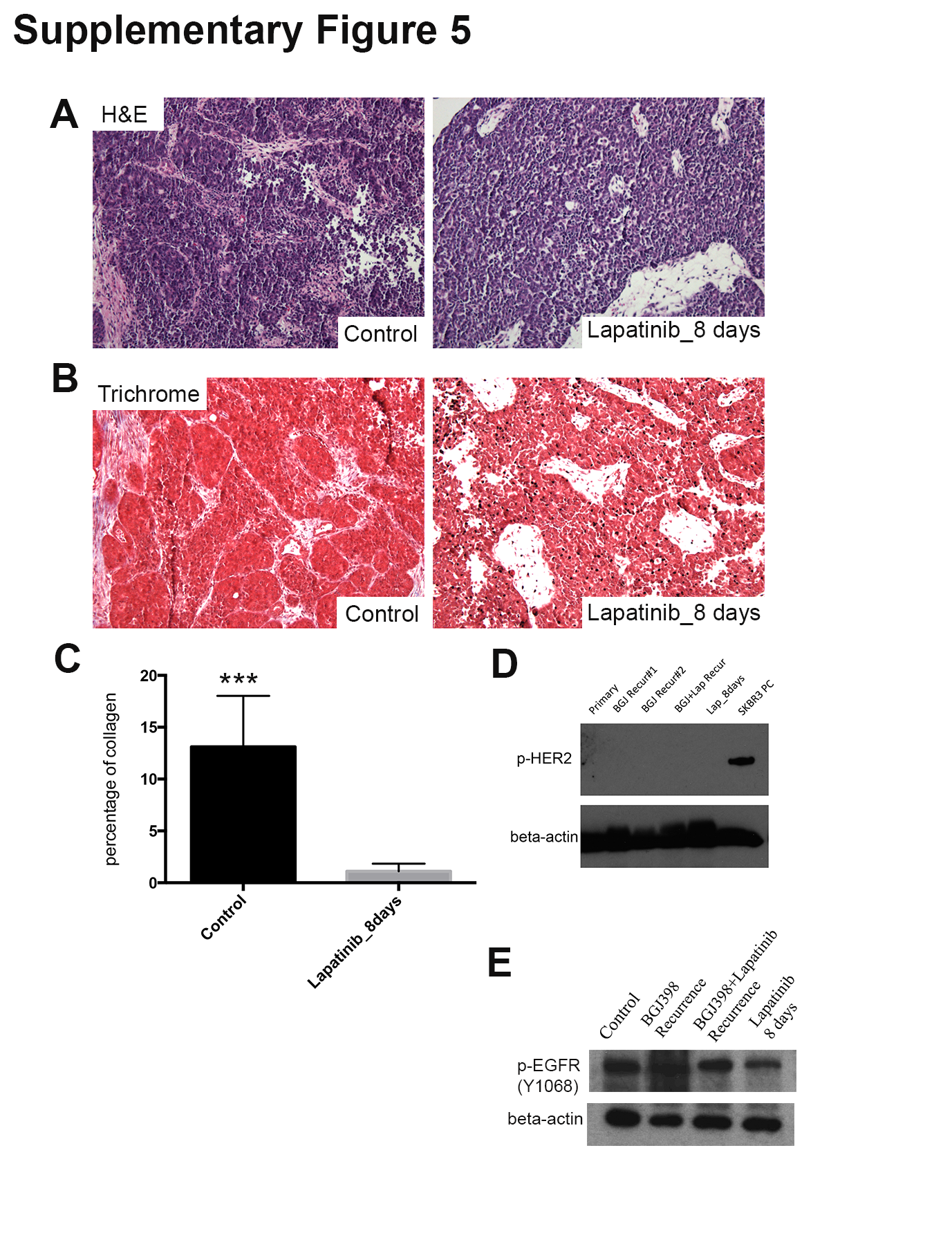

Supplement: Additional file 8: Figure S7. — Lapatinib treatment reduces collagen expression in the tumor stroma. A. H&E of tumors from control or treatment groups with 8 days of lapatinib treatment. B. Trichrome staining of tumors from control or treatment groups with 8 days of lapatinib treatment. C. Quantification of percentage of collagen in tumors from control and 8-day lapatinib treatment group; ***p < 0.001. Values are shown as mean ± SEM. D. Immunoblot analysis of p-ErbB2 in primary Wnt1/iR1 tumors, recurrent tumors arising from BGJ398 treatment or BGJ398 + lapatinib treatment, and tumors treated with lapatinib for 8 days. SKBR3 cell lysate was used as a positive control for p-ErbB2. Beta-actin was used as a loading control. E. Immunoblot analysis of p-EGFR (Y1068) in Wnt1/iR1 tumors treated with lapatinib for 8 days, as well as recurrent tumors arising from BGJ398 treatment or BGJ398 + lapatinib treatment, as compared to control group treated with vehicle. Beta-actin was used as a loading control. (TIF 32982 kb) [file 13058_2015_649_MOESM8_ESM.tif]

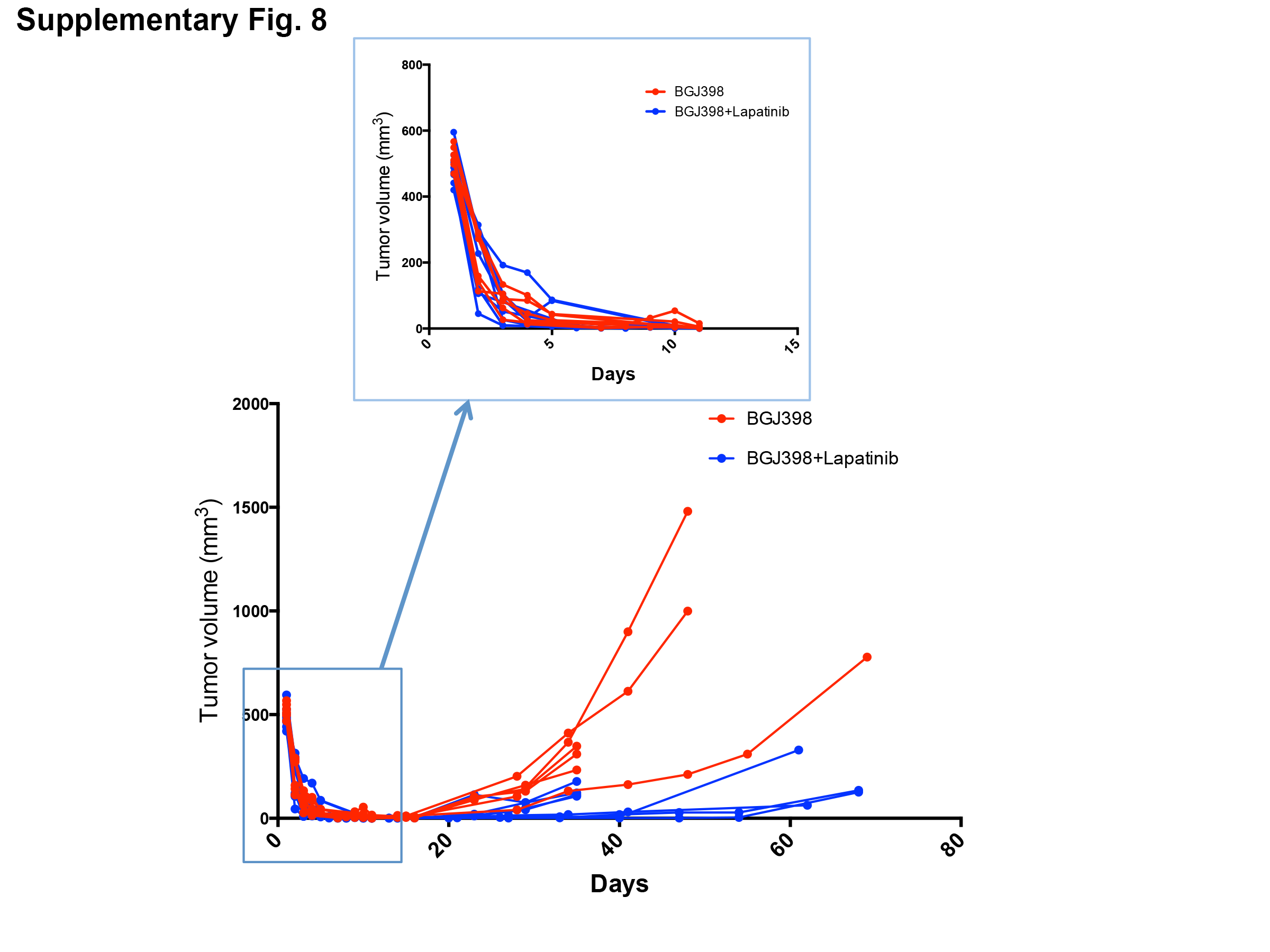

Supplement: Additional file 9: Figure S8. — Individual plots of tumor regression and recurrence in the BGJ398 and BGJ398 and lapatinib treatment groups. Six mice in the single BGJ398 treatment group, eight mice in the combined BGJ398 and lapatinib treatment group, 14 mice in total. (TIF 13485 kb) [file 13058_2015_649_MOESM9_ESM.tif]
